# Species limits and recent diversification of *Cerradomys* (Sigmodontinae: Oryzomyini) during the Pleistocene

**DOI:** 10.7717/peerj.13011

**Published:** 2022-04-22

**Authors:** Camilla B. Di-Nizo, Elkin Y. Suárez-Villota, Maria José J. Silva

**Affiliations:** 1 Laboratório de Ecologia e Evolução, Instituto Butantan, São Paulo, São Paulo, Brazil; 2 Leibniz Institute for the Analysis of Biodiversity Change, Zoological Research Museum A. Koenig, Bonn, Germany; 3 Instituto de Ciencias Naturales, Facultad de Medicina Veterinaria y Agronomía, Universidad de las Américas, Concepción, Chile

**Keywords:** Species delimitation, Coalescent models, Cytotaxonomy, Cryptic species, Molecular systematics, Cricetidae, Neotropics

## Abstract

*Cerradomys* is a genus of the tribe Oryzomyini with eight species currently recognized, and a controversial taxonomy. These species are mainly distributed in the South America dry diagonal, but some species extend into Atlantic Forest, reaching the coastal sandy plains known as Restingas. This study aimed to address species limits and patterns of diversification of *Cerradomys* species. For this purpose, we performed cytogenetic and molecular analyses (phylogeny, coalescent species delimitation, barcoding, and divergence times estimation) using multiple mitochondrial and nuclear markers on a comprehensive sampling, representing all nominal taxa reported so far. Chromosomal information was a robust marker recognizing eight *Cerradomys* species. Reciprocal monophyly was recovered for all the species, except for *C. subflavus*. These results together with coalescent analyses recovered eight species as the most congruent species delimitation scenario for the genus (mean C_*tax*_: 0.72). Divergence time estimates revealed that *Cerradomys*’ diversification occurred about 1.32 million years ago (Mya) during the Pleistocene. Although our results conservatively support the eight *Cerradomys* species described so far, different lines of evidence suggest that *C. langguthi* and *C. subflavus* could potentially be species-complexes. We discussed this scenario in the light of multiple evolutionary processes within and between species and populations, since *Cerradomys* comprises a species group with recent diversification affected by Pleistocene climatic changes and by the complex biogeographic history of South America dry diagonal. This work supports that the diversity of *Cerradomys* is underestimated and reiterates that interdisciplinary approaches are mandatory to identify small rodent species properly, and to unhide cryptic species.

## Introduction

The tribe Oryzomyini is widely distributed from Tierra del Fuego (southern South America) to the southeastern United States, on the Galapagos Archipelago, and on Trinidad and Tobago and is undoubtedly the most diverse Sigmodontinae radiation, encompassing nearly one third of the diversity of this subfamily (Weksler, 2015; Pardiñas et al., 2017). This diversity is reflected in morphological, ecological, molecular and chromosomal variations, leading to a complex taxonomic history.

The genus *Oryzomys*, for example, came to consist of almost half of all Oryzomyini species and previous phylogenetic analyses recovered it as paraphyletic (Myers, Lundrigan & Tucker, 1995; Bonvicino & Moreira, 2001; Weksler, 2003). To assess the monophyly of oryzomyine rodents, Weksler, Percequillo & Voss (2006), based on morphological and molecular data, described ten new genera for species and species groups formerly referred to as *Oryzomys.* A combination of different delimitation criteria together with fieldwork and taxonomic revision efforts led to an increase in the number of recognized species and even description of new Oryzomyini genera in the last decade (Percequillo, Weksler & Costa, 2011; Brito et al., 2020; Hurtado, 2021; Semedo et al., 2021), reflecting the high and hide rodent diversity of this group (Burgin et al., 2018; D’Elía, Fabre & Lessa, 2019). At present, 30 extant genera compose this tribe (Pardiñas et al., 2017; Brito et al., 2020; Percequillo et al., 2021).

*Cerradomys* Weksler, Percequillo & Voss, 2006 was formerly included in the *Oryzomys subflavus* group and was considered monotypic for a long time (Weksler, Percequillo & Voss, 2006). Cytogenetic studies performed during the 1980s and 1990s were the first indications that “*Oryzomys subflavus*” could contain more than one species, since four different karyotypes were attributed to a single taxonomic entity (Maia & Hulak, 1981; Almeida & Yonenaga-Yassuda, 1985; Svartman & Almeida, 1992; Bonvicino, Otazu & Borodin, 1999).

Interdisciplinary approaches, which included morphology and molecular phylogeny, later confirmed that the taxon was not monotypic (Bonvicino & Moreira, 2001). Currently, eight species are described: *C. akroai* Bonvicino, Casado & Weksler, 2014, *C. goytaca* Tavares, Pessôa & Gonçalves, 2011, *C. langguthi* Percequillo, Hingst-Zaher & Bonvicino, 2008, *C. maracajuensis* (Langguth & Bonvicino, 2002), *C. marinhus* (Bonvicino, 2003), *C. scotti* (Langguth & Bonvicino, 2002), *C. subflavus* (Wagner, 1842) and *C. vivoi* Percequillo, Hingst-Zaher & Bonvicino, 2008.

*Cerradomys’* representatives inhabit mainly the open vegetation areas in South America, from northeastern Brazil to southeastern Bolivia, reaching southern Peru and northwestern Paraguay (Carleton & Musser, 2005; Tavares, Pessôa & Gonçalves, 2011; Percequillo, 2015). Three species (*C. langguthi, C. subflavus* and *C. vivoi*) alongside their distribution in the Cerrado and Caatinga domains, can penetrate the Atlantic Forest, and *C. goytaca* is the only species endemic to the sandy coastal soils of Restinga formation (Percequillo, Hingst-Zaher & Bonvicino, 2008; Tavares, Pessôa & Gonçalves, 2011). Although some species have allopatric distribution (*i.e.*, *C. goytaca*), other species are widely distributed in open habitats of the Cerrado, such as *C. scotti*, that may be found in sympatry with *C. macarajuensis*, *C. marinhus,* and *C. subflavus*.

*Cerradomys subflavus, C. vivoi,* and *C. goytaca* have not been recovered as reciprocally monophyletic using single-locus molecular phylogeny, suggesting that they might be conspecific (Bonvicino, Casado & Weksler, 2014). However, Tavares, Pessôa & Seuánez (2016) based on morphometric data, and more recently Di-Nizo, Ferguson-Smith & Silva (2020) based on comparative chromosome analyses, suggested that these three lineages should be recognized as distinct species.

Thus, different approaches proved to be essential to understand this complex group, since different methods have provided incongruent results on species limit. In addition, until now, *Cerradomys* species were not subject to studies with enough specimens and different loci to allow the differentiation of population to species level. Herein, we combined cytogenetic and several molecular methods (multi-locus phylogenetic inference, DNA barcoding, coalescent-based species delimitation), together with distribution data in a large sample comprising the eight species described, to address species limits and the phylogenetic relationships of *Cerradomys*, according to multiple approaches by congruence (Padial et al., 2010). Finally, we estimated divergence times of *Cerradomys* species to assess its evolutionary history and address hypotheses of *tempo* and mode of evolution.

## Material & Methods

### Samples

**Figure 1:**
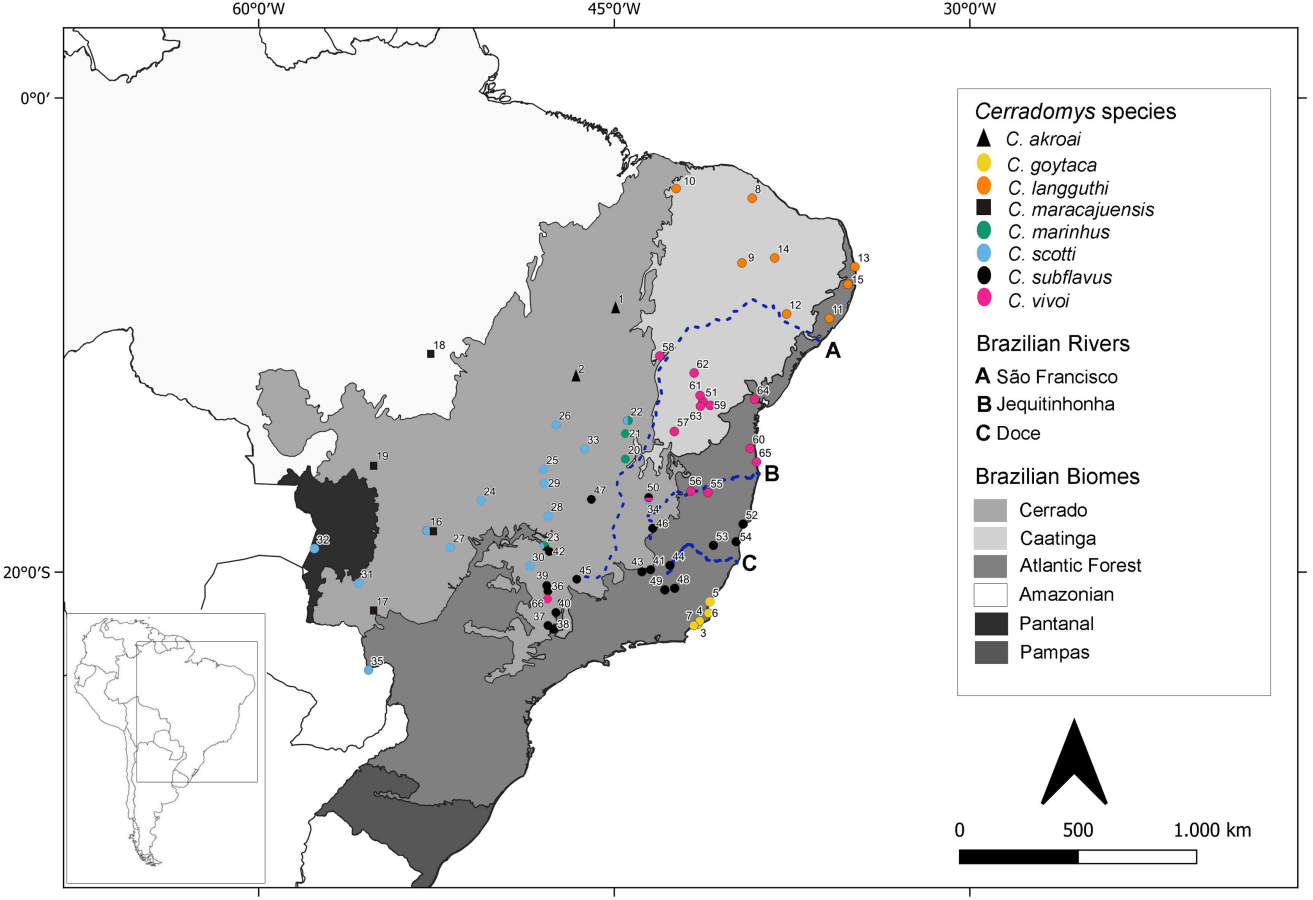
Geographic distribution of *Cerradomys* studied in this work plus localities from sequences extracted from GenBank. Numbers correspond to localities in Table S1.

Ninety-four individuals previously identified as *Cerradomys* sp. were analyzed under molecular approaches (Table S1 - in bold). From these specimens, 35 have cytogenetic information obtained in this study (26 individuals) or by Di-Nizo, Ferguson-Smith & Silva (2020) (nine individuals) (Table S1). Samples were collected in 41 localities from 10 Brazilian States. A map containing the collecting localities encompassing animals studied in the present study plus localities from samples downloaded from GenBank is shown in Fig. 1, totalizing 65 localities from Brazil and one from Paraguay.

Surveys were carried out under license numbers ICMBio 11603-1 and 24003-4 of the Instituto Chico Mendes de Conservação da Biodiversidade. Some specimens were captured by collaborators under their respective licenses. Animals were euthanized following the guidelines of the American Society of Mammalogists (Sikes, 2016) and under permission of Instituto Butantan Ethics Committee (CEUAIB 1151/13). Skins, skulls, and partial skeletons were deposited in Brazilian Museums and Universities according to Table S1.

### Cytogenetics

Metaphases were obtained *in vivo* from spleen and bone marrow (Ford & Hamerton, 1956) or *in vitro* from fibroblast cell culture (Freshney, 1986).

Conventional Giemsa staining was used to determine the diploid number (2n) and the number of autosome arms (FN). To identify properly sex chromosomes and homologues, CBG- and GTG-banding were performed following Sumner (1972) and Seabright (1971), respectively (data not shown). Metaphases were captured either with visible light on Axioskop 40 microscope (Carl Zeiss) using AxioVision software or with Zeiss Axiphot microscope (Carl Zeiss) using Ikaros Metasystems software.

### DNA extraction, amplifications and sequencing

DNA was extracted from liver or muscle using Chelex100 (Walsh, Metzger & Higuchi, 1991). Partial cytochrome *b* (cyt-*b*), cytochrome oxidase subunit 1 (COI), interphotoreceptor first exon of retinoid binding protein (IRBP), and intron 7 of *β*-fibrinogen (i7FBG) sequences were amplified using Polymerase Chain Reaction (PCR) - primers and conditions are presented in Table S2. Master-Mix for PCR, purification, and PCR products visualization were performed according to Suárez-Villota et al. (2018).

Sequencing was performed with BigDye (Applied Biosystesm, Waltham, MA, USA) in an ABI PRISM 3100 Genetic Analyzer (Applied Biosystesm, Waltham, MA, USA). Electropherograms were visualized and aligned with Geneious 7.1.7 (GeneMatters Corp., San Francisco, CA, USA) (Kearse et al., 2012) using MUSCLE (Edgar, 2004). Sequences were submitted to a comparative similarity search on BLAST (*Basic Local Alignment Search Tool*) before the alignment. For nuclear sequences, double peaks were codified in both strands as ambiguous sites according to IUPAC code. Sequences submitted and downloaded from GenBank are listed in the Table S1.

### Phylogenetic reconstruction

The phylogenies were reconstructed based on Bayesian Inference (BI) and Maximum Likelihood (ML). Seven matrices were constructed and the number of base pairs, terminal taxa and the analyses performed for each matrix are compiled in Table S3. Outgroup for molecular phylogeny analyses was composed of *Oligoryzomys flavescens* (Waterhouse, 1837)*, Holochilus sciureus* (Wagner, 1842) and *Calomys tener* (Winge, 1887) (*sensu* (Weksler, Percequillo & Voss, 2006)) and of different species of the subfamily Sigmodontinae to employ fossil calibration points for molecular dating (Table S4).

The best-fit partitioning schemes and models of nucleotide substitution were selected using the Bayesian information criterion (BIC) implemented in PartitionFinder 2.1.1 (Lanfear et al., 2016). ML was carried out with GARLI 0.951 (Bazinet, Zwickl & Cummings, 2014). Statistical support for the nodes was estimated by nonparametric bootstrapping (Felsenstein, 1985), with 1000 pseudoreplicates. Bayesian inference was carried out in MrBayes 3.2.6 (Ronquist & Huelsenbeck, 2003). Markov chains were started from a random tree and run for 1.0 ×10^7^ generations with sampling every 1000th generation. The stationary phase was checked using Tracer 1.6 (Rambaut et al., 2014). Sample points - before the plateau phase - were discarded as burn in, and the remaining trees were combined to find the maximum *a posteriori* estimated probability of the phylogeny. Branch supports were estimated with Bayesian posterior probabilities. Two simultaneous analyses were performed to ensure convergence of the topologies.

### Evolutionary distance and Automatic Barcode Gap Discovery (ABGD)

Evolutionary genetic distances, using Kimura 2-parameter (K2P, Kimura, 1980) molecular evolution model for cyt-*b* and COI data sets were measured using MEGA 7 (Kumar, Stecher & Tamura, 2016).

For barcoding, we carried out the Automatic Barcode Gap Discovery (ABGD) analysis, which groups the input sequences into several hypothetical species by calculating all pairwise distances in the data set (Puillandre et al., 2012). The ABGD analysis was performed online, using three different distance metrics: K2P (Kimura, 1980), Jukes and Cantor (JC69) (Jukes & Cantor, 1969), and simple distance (p-distance) (Nei & Kumar, 2000). The parameters used were Pmin (0.001) and Pmax (0.2), relative gap width (*X* = 1.5) and the other parameters set to default values.

### Coalescent-based species delimitation methods

For single-locus analyses, Bayesian implementation of Poisson Tree Processes (bPTP) (Fujisawa & Barraclough, 2013; Zhang et al., 2013) and General Mixed Yule Coalescent model (GMYC) (Pons et al., 2006) were performed. Since most specimens available in GenBank have only cyt-*b* and too many gaps could affect the results (Pons et al., 2006; Fujisawa & Barraclough, 2013), two analyses were performed: (i) with the cyt-*b* matrix (that included sequences generated in this study plus sequences from GenBank) and (ii) with the mitochondrial matrix (cyt-*b* + COI – with sequences from the present study). For bPTP analysis, the BI topology was used as the input on the web server of the Exelixis Lab (http://species.h-its.org/ptp) (Zhang et al., 2013). To perform GMYC analysis, the BI topology was ultrametrized in Mesquite (version 3.2) (Maddison & Maddison, 2017). The tree was implemented in GMYC web service (http://species.h-its.org/gmyc/) assuming a single threshold (Pons et al., 2006; Fujisawa & Barraclough, 2013; Zhang et al., 2013).

For multi-locus species delimitation, we performed two coalescent-based analyses: Species Tree Estimation using Maximum Likelihood (STEM; Kubatko, Carstens & Knowles, 2009) and Bayesian Phylogenetics and Phylogeography program (BPP; Yang & Rannala, 2010; Yang, 2015).

For STEM analysis, we estimate ML scores for each species tree in STEM v2.0 (Kubatko, Carstens & Knowles, 2009) and evaluate the best scenario following Harrington & Near (2012). We assigned individuals to a series of species categories (from two to 69 species) using chromosome data, cyt-*b* monophyletic groups, unilocus species delimitation (mPTP and GMYC cyt-*b*) results, and geographical distribution (we test allopatric distribution in the case of *C. goytaca* and *C. subflavus* since they were not reciprocally monophyletic).

For BPP, the population size parameters (*θ*s) were assigned the inverse-gamma prior IG (3, 0.02), with mean 0.01 in BPP software version 4.3.8 (Yang & Rannala, 2010; Yang, 2015). The divergence time at the root of the species tree (*τ*0) was assigned the inverse-gamma prior IG (3, 0.16), with mean 0.08 while the other divergence time parameters were specified by the uniform Dirichlet distribution (Yang & Rannala, 2010: equation 2). An initial A00 analysis was run for estimation of the parameters of population sizes (*θ*s) and species divergence times (*τ*s). Subsequently, we used A11 mode, which joint species delimitation and species tree inference of unguided species delimitation (speciesdelimitation = 1, speciestree = 1) with *θ* and *τ* priors estimated from the initial analysis, as well as using BPP’s built-in function to re-estimate theta during the simulation to avoid any existing taxonomic bias. We run each analysis twice for a total of 500,000 MCMC simulations guided by a “burn-in” of 50,000 iterations extra to confirm consistency among results. Since BPP attempts to merge different populations into one species but never tries to split one population into multiple species, we start using the scenario with the highest number of species (69 species proposed by GMYC cyt-*b* analyses) and its species tree estimated by STEM, as prior. According to a conservative approach, we considered only speciation events simultaneously supporting probabilities equal to 1.0 for all combinations of priors for species delimitation.

To explore the congruence inferred by the different species delimitation approaches, the taxonomic index of congruence (C_*tax*_) was estimated according to Miralles & Vences (2013). In this analysis, we included all coalescent species delimitation approaches, groups inferred by cytogenetic data, ABGD analysis, and available morphological data (Langguth & Bonvicino, 2002; Bonvicino, 2003; Percequillo, Hingst-Zaher & Bonvicino, 2008; Tavares, Pessôa & Gonçalves, 2011; Bonvicino, Casado & Weksler, 2014).

### Molecular dating

Ages of clades and taxa were estimated with the concatenated multi-locus matrix and sequences from other 12 Sigmodontinae species used as constraints for fossil calibration (see Table S3). We used the same models and partitions obtained in PartitionFinder 2.1.1 (Lanfear et al., 2016) implemented in the phylogenetic analyses.

Divergence times were estimated using a Bayesian MCMC approach implemented in BEAST 1.8.3 (Drummond et al., 2012). An uncorrelated lognormal relaxed molecular clock with a Birth and Death incomplete sampling tree prior and random starting tree were implemented. The ages of the clades were constrained as log-normally distributed priors (Ho & Phillips, 2009) with offsets adjusted to accommodate fossil dates (clade minimum age) within the first 5% percentile of a log-normal distribution with mean 0.01 and standard deviation 1.0.

Seven calibration points based on fossil records were used as minimum constraints following Pardiñas, D’Elía & Ortiz (2002), Voglino & Pardiñas (2005) and Ronez et al. (2021): (i) crown age of the genus *Neotoma* (offset: 4.57 Mya); (ii) crown age of the genus *Sigmodon* (2.5 Mya); (iii) crown of the genus *Reithrodon* (offset: 3.8 Mya); (iv) crown Akodontini (offset: 3.8 Mya); (v) crown Phyllotini (offset: 4.3 Mya); (vi) crown age of the genus *Graomys* (offset: 3.8 Mya) and (vii) crown of the genus *Oligoryzomys* (offset: 0.8 Mya). Four analyses were run each one with 10 million generations or until convergence (until the parameters of effective sample size—ESS were greater than or equal to 200), sampled every 1000 generations. Stationarity of the MCMC chain, ESS parameters, and posterior intervals spanning the 95% highest posterior density (HPD) were assessed using Tracer1.6 (Rambaut et al., 2014).

## Results

### Cytogenetics

**Figure 2:**
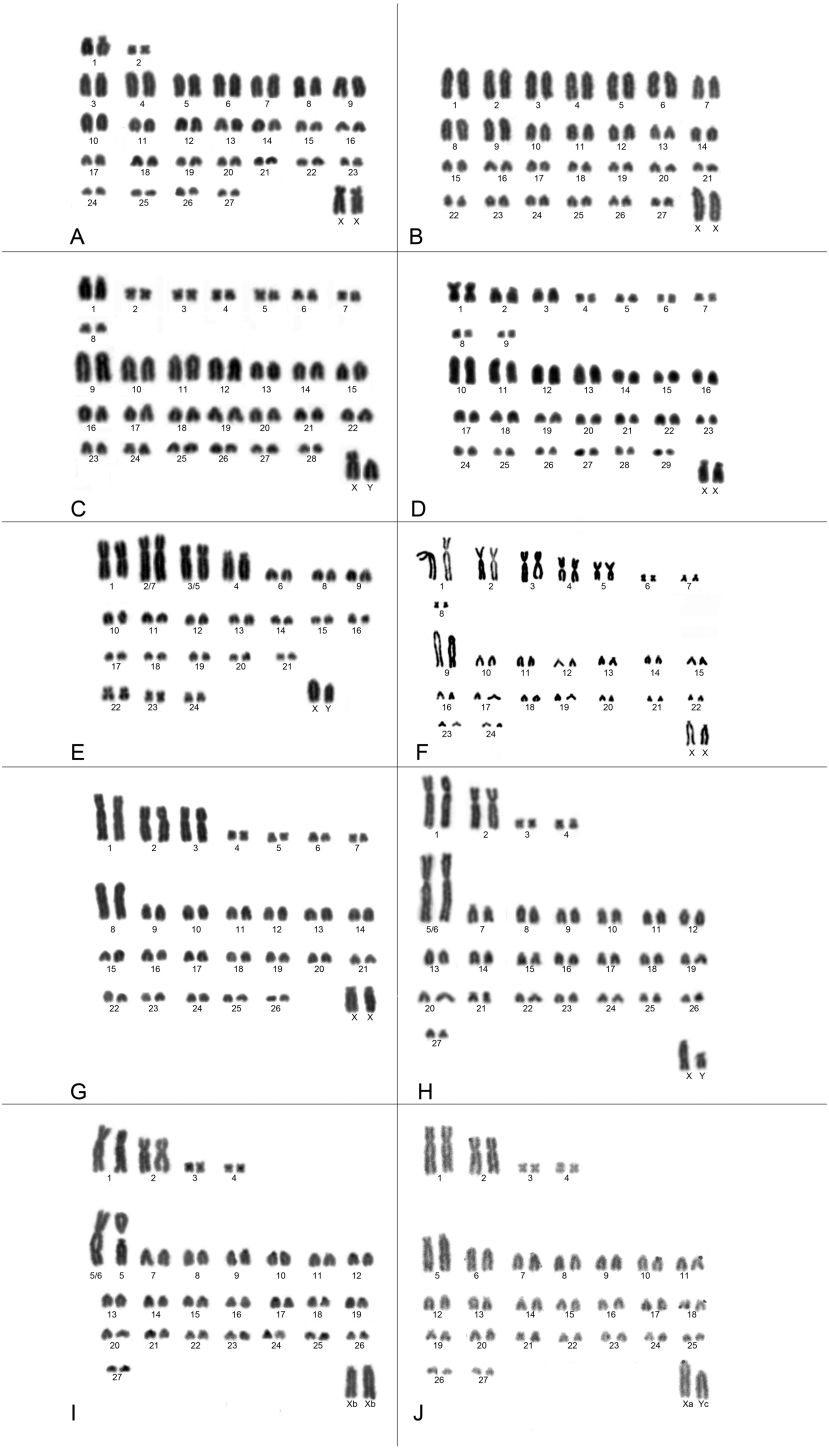
Karyotypes of *Cerradomys* species. (A) *C. maracajuensis* –2n = 56, FN = 58; (B) *C. marinhus* –2n = 56, FN = 54; (C) *C. scotti* –2n = 58, FN = 72; (D) *C. akroai* –2n = 60, FN = 76; (E) *C. langguthi* –2n = 46, FN = 56; (F) *C. vivoi* –2n = 50, FN = 64; (G) *C. goytaca* –2n = 54, FN = 66; (H) *C. subflavus* karyotype (i) –2n = 54, FN = 62; (I) *C. subflavus* karyotype (ii) –2n = 55, FN = 63 and (J) *C. subflavus* karyotype (iii) –2n = 56, FN = 64.

Karyotypes were associated to eight names following cytogenetic data reported previously in the literature and in accordance with the position of samples recovered in the molecular phylogeny, which included the holotype/paratype: (i) *C. maracajuensis -* 2n = 56, FN = 58 (Fig. 2A); (ii) *C. marinhus -* 2n = 56, FN = 54 (Fig. 2B); (iii) *C. scotti -* 2n = 58, FN = 72 (Fig. 2C); (iv) *C. akroai -* 2n = 60, FN = 76 (Fig. 2D); (v) *C. langguthi -* 2n = 46, FN = 56 (Fig. 2E); (vi) *C. vivoi* - 2n = 50, FN = 64 (Fig. 2F); (vii) *C. goytaca* - 2n = 54, FN = 66 (Fig. 2G) and (viii) *C. subflavus* –2n = 56-54, FN = 64-62 (Figs. 2H–2J). The latter showed three different diploid numbers: 2n = 54, FN = 62 (Fig. 2H); 2n = 55, FN = 63 (Fig. 2I) and 2n = 56, FN = 64 (Fig. 2J) and the differences were related to Robertsonian rearrangements involving pairs 5 and 6. Karyotype with 2n = 54 showed one very large metacentric pair that corresponds to pairs 5 and 6. Karyotype with 2n = 55 showed a single very large submetacentric (5/6), one subtelocentric (5) and one acrocentric (6) chromosomes. Karyotype with 2n = 56 showed pair 5 subtelocentric and pair 6 acrocentric.

### Phylogenetic reconstruction

**Figure 3:**
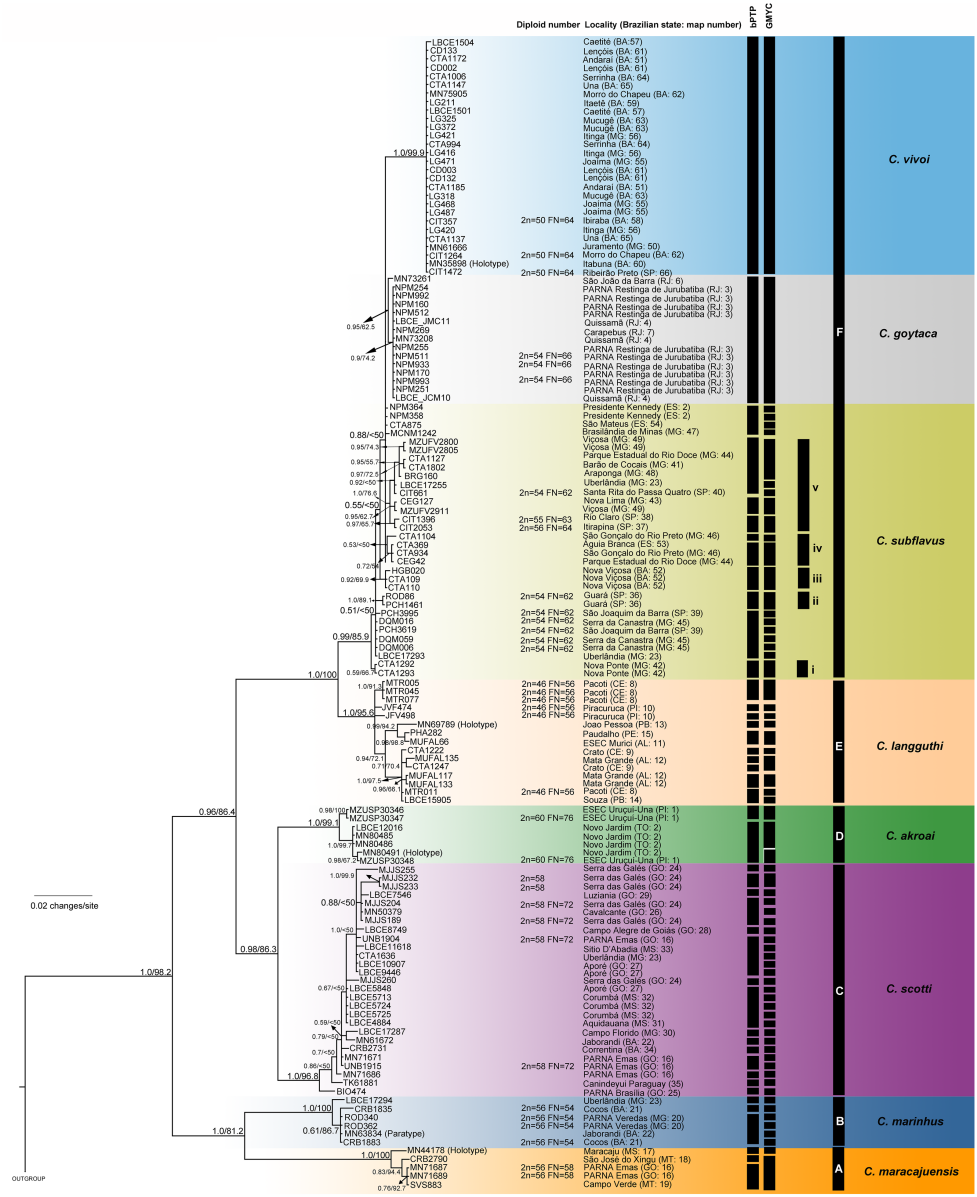
Bayesian Inference (BI) tree based on mitochondrial gene cyt-*b*. Numbers in the nodes indicate BI posterior probability (above 0.50) and ML bootstrap support (above 50), respectively. Black bars from the left to the right indicate results of single-locus coalescent-based species delimitation (bPTP and GMYC), subclades and clades recovered by BI and ML, respectively.

**Figure 4:**
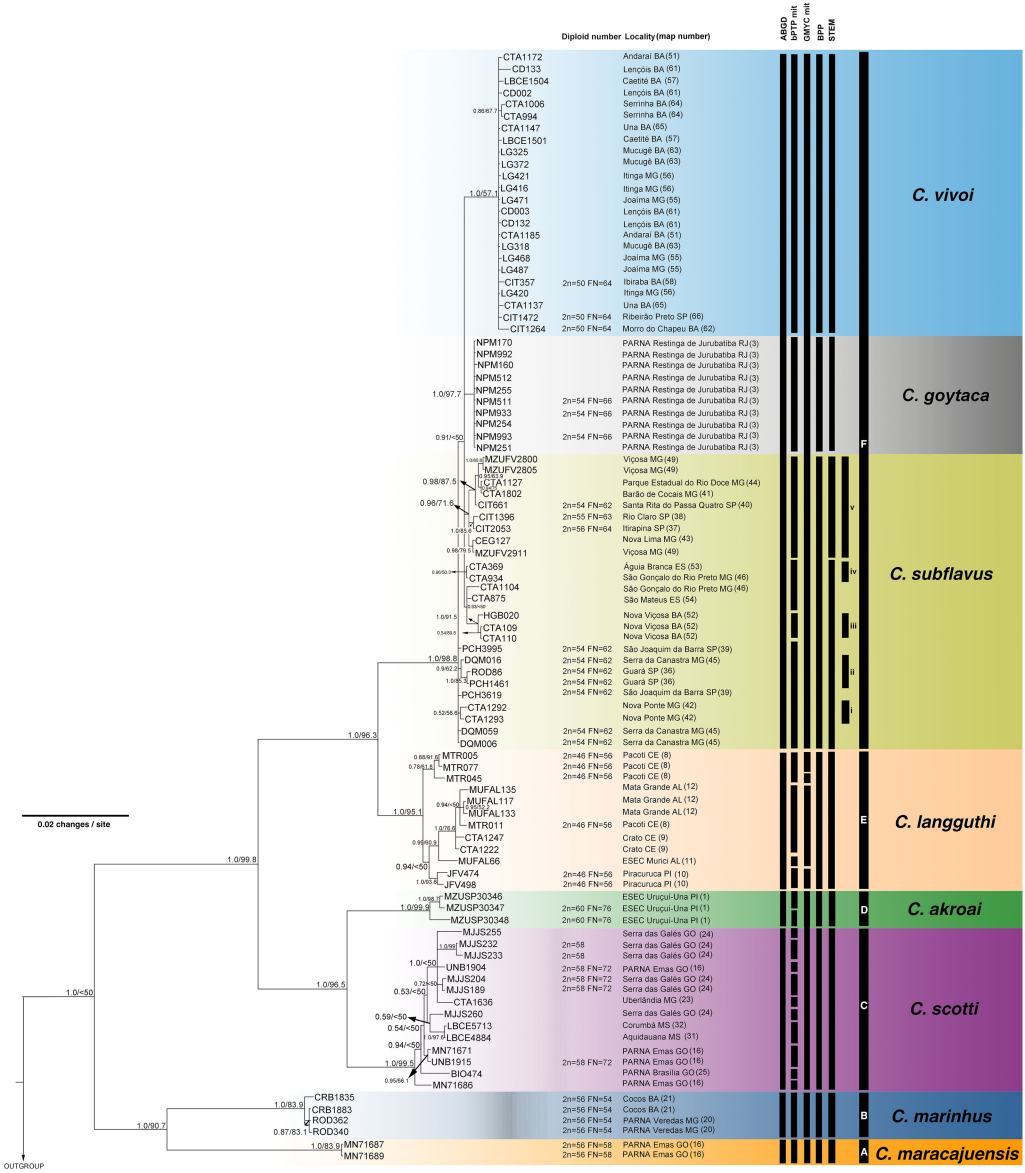
Bayesian Inference (BI) tree based on concatenated data set (cyt-*b*, COI, IRBP and i7FBG). Numbers in the nodes indicate BI posterior probability (above 0.50) and ML bootstrap support (above 50), respectively. Black bars from the left to the right indicate results of ABGD, bPTP and GMYC with mitochondrial data set, multi-locus coalescent-based species delimitation BPP and STEM and subclades and clades recovered by BI and ML, respectively.

The best-fit models selected for each gene is shown in Table S2, and these models and partition schemes were also used for concatenated multi-locus analyses. Phylogenetic reconstructions using cyt-*b* and concatenated multi-locus data sets recovered *Cerradomys* as monophyletic [cyt-*b*: 1.0 of Bayesian posterior probability (PP)/98.2 of maximum likelihood bootstrap support (ML), multi-locus: 1.0PP/<50ML] and the same topology, with six main clades (Clades A–F, Figs. 3 and 4), as follows: **Clade A** represented by *C. maracajuensis*, including the holotype (cyt-*b*: 1.0PP/ 100ML, multi-locus: 1.0PP/83.9 ML); **Clade B** by *C. marinhus*, including the paratype (cyt-*b*: 1.0PP/100ML, multi-locus: 1.0PP/83.9ML); **Clade C** by sequences from individuals treated as *C. scotti* (cyt-*b*: 1.0PP/96.8ML, multi-locus: 1.0PP/99.5ML); **Clade D** by *C. akroai*, also including sequence of the holotype (cyt-*b*: 1.0PP/99.1ML, multi-locus: 1.0PP/99.9ML); **Clade E** is composed of *C. langguthi,* including sequence of the holotype (cyt-*b*: 1.0PP/95.6ML, multi-locus: 1.0PP/95.1ML), and **Clade F** grouped sequences assigned to *C. vivoi* (including the holotype), *C. goytaca*, and *C. subflavus* (cyt-*b*: 0.99PP/85.9ML, multi-locus: 1.0PP/98.8ML) (Figs. 3 and 4).

*Cerradomys maracajuensis* from Cerrado of Goiás, Mato Grosso do Sul and transition areas of Cerrado and Amazonian Forest of Mato Grosso State and *C. marinhus* from the Cerrado of Minas Gerais and Bahia states (Clades A and B) were recovered as sister clades with high to moderate support (cyt-*b*: 1.0PP/81.2ML, multi-locus: 1.0PP/90.7ML). These species were recovered as the sister group to the remaining *Cerradomys* species (Clades C-F; Figs. 3 and 4).

*Cerradomys scotti* (Clade C) included sequences from the Brazilian states of Goiás, Minas Gerais, Bahia, and Mato Grosso do Sul and it is closely related to *C. akroai* (Clade D), composed of individuals from the Cerrado of Piauí and Tocantins states (cyt-*b*: 0.98PP/86.3ML, multi-locus: 1.0PP/96.5ML). This clade composed of *C. scotti* and *C. akroai* is recovered as the sister group to clades E and F (cyt-*b*: 0.96PP/86.4ML, multi-locus: 1.0PP/99.8ML) from Eastern Brazil (Figs. 3 and 4).

*Cerradomys langguthi* (Clade E, encompassing individuals from Northeast Brazil, distributed in Atlantic Forest, Cerrado, and Brejos—mountain ranges of humid forests in areas of Caatinga—and Clade F were recovered as a monophyletic group with high support (cyt-*b*: 1.0PP/100ML, multi-locus: 1.0PP/96.3ML).

Clade F has two main monophyletic clades, one composed of *C. vivoi* (distributed in Cerrado, Caatinga, Atlantic Forest and transitional areas of Minas Gerais, São Paulo and Bahia states) (cyt-*b*: 1.0PP/99.9ML, multi-locus: 1.0PP/57.1ML) and the other composed of *C. goytaca*, endemic to Restingas (cyt-*b*: 0.95PP/62.5ML, multi-locus: 1.0PP/97.7ML) (Figs. 3 and 4). For the cyt-*b* analyses, the set of sequences downloaded from GenBank referred to as *C. goytaca* from Presidente Kennedy, Espírito Santo state, were recovered polyphyletic (Fig. 3).

Within Clade F, sequences referred to *C. subflavus* from Atlantic Forest and Cerrado were recovered into five main subclades that differed slightly between cyt-*b* and multi-locus analyses in their composition, since cyt-*b* matrix was composed not only of sequences obtained in this work, but also sequences from GenBank. The cyt-*b* analyses showed subclade (i) with samples from Nova Ponte, Minas Gerais state (0.59PP/ 66.7ML); (ii) two samples from Guará, São Paulo state (1.0PP/ 89.1 ML); (iii) three samples from Nova Viçosa, Bahia state (0.92 PP/ 69.9ML); (iv) four samples from Minas Gerais and Espírito Santo states (0.53PP/ <50ML) and (v) 11 samples from São Paulo and Minas Gerais states (1.0PP/ 76.6 ML) (Fig. 3). Multi-locus analyses recovered the same subclades (i, iii and v); subclade (ii) was composed of the same two sequences from Guará, São Paulo state plus one sequence from Serra da Canastra, Minas Gerais state (0.9PP / 62ML) and subclade (iv) only one sample from Minas Gerais state and one from Espírito Santo state (0.96PP/ 50.3 ML) (Fig. 4). Other samples assigned to *C. suflavus* from São Paulo, Minas Gerais, and Espírito Santo states were not recovered in any of these five subclades and exhibited a polyphyletic pattern within Clade F (Fig. 3).

All the other single-locus analyses recovered *Cerradomys* as monophyletic (COI: 0.98PP/85.8ML; IRBP: 1.0PP/ 86.3ML and i7FBG: 1.0PP/ 99.7ML) and provided similar topology to the cyt-*b* and multi-gene analyses, although with lower support (Figs. S1–S3). Topological disagreements were observed mainly in the low-supported branches.

### Evolutionary distance and Automatic Barcode Gap Discovery (ABGD)

Intraspecific distances of cyt-*b* varied from 0 (zero) in *C. vivoi* to 1.2% in *C. langguthi*. The lowest interspecific distance was observed between *C. subflavus* and *C. goytaca* (0.7%) and the highest was observed between *C. maracajuensis* and *C. goytaca* (15%) (Table 1).

**Table 1:** Distance and standard deviation obtained for *Cerradomys* with K2P model of evolution based on cyt-*b* data set. Diagonal in bold represents intraspecific distance.

		1	2	3	4	5	6	7	8
1	*C. maracajuensis*	**0.006 ± 0.002**							
2	*C. marinhus*	0.105 ± 0.013	**0.004 ± 0.001**						
3	*C. scotti*	0.119 ± 0.014	0.113 ± 0.013	**0.006 ± 0.001**					
4	*C. akroai*	0.132 ± 0.015	0.112 ± 0.014	0.053 ± 0.009	**0.005 ± 0.002**				
5	*C. langguthi*	0.146 ± 0.016	0.138 ± 0.015	0.089 ± 0.012	0.094 ± 0.012	**0.012 ± 0.003**			
6	*C. vivoi*	0.14 ± 0.016	0.124 ± 0.015	0.09 ± 0.012	0.096 ± 0.012	0.052 ± 0.008	**0.000 ± 0.000**		
7	*C. goytaca*	0.151 ± 0.017	0.138 ± 0.016	0.091 ± 0.012	0.099 ± 0.013	0.041 ± 0.007	0.022 ± 0.006	**0.001 ± 0.001**	
8	*C. subflavus*	0.148 ± 0.017	0.136 ± 0.015	0.088 ± 0.012	0.098 ± 0.013	0.04 ± 0.007	0.023 ± 0.006	0.007 ± 0.002	**0.006 ± 0.002**

Intraespecific distances of COI varied from 0 (zero) in *C. maracajuensis* and *C. goytaca* to 1.2% in *C. langguthi*. Interespecific distance varied from 0.9% (*C. subflavus* and *C. goytaca*) to 14.4% (*C. maracajuensis* and *C. vivoi*) (Table 2). The ABGD analyses recovered six candidate species (Fig. 4), although the extreme *p* values yielded between 3 and 32 candidate species (Table 3, Fig. S4).

**Table 2:** Distance and standard deviation obtained for *Cerradomys* with K2P model of evolution based on COI data set. Diagonal in bold represents intraspecific distance.

		1	2	3	4	5	6	7	8
1	*C. maracajuensis*	**0.000 ± 0.000**							
2	*C. marinhus*	0.091 ± 0.013	**0.001 ± 0.001**						
3	*C. scotti*	0.137 ± 0.022	0.106 ± 0.012	**0.006 ± 0.002**					
4	*C. akroai*	0.120 ± 0.011	0.113 ± 0.022	0.046 ± 0.017	**0.004 ± 0.002**				
5	*C. langguthi*	0.125 ± 0.018	0.097 ± 0.018	0.063 ± 0.015	0.037 ± 0.017	**0.012 ± 0.003**			
6	*C. vivoi*	0.144 ± 0.016	0.109 ± 0.018	0.090 ± 0.019	0.069 ± 0.014	0.043 ± 0.012	**0.001 ± 0.001**		
7	*C. goytaca*	0.140 ± 0.020	0.105 ± 0.022	0.091 ± 0.006	0.073 ± 0.018	0.056 ± 0.015	0.015 ± 0.017	**0.000 ± 0.000**	
8	*C. subflavus*	0.139 ± 0.016	0.106 ± 0.005	0.091 ± 0.004	0.072 ± 0.009	0.050 ± 0.021	0.012 ± 0.017	0.009 ± 0.011	**0.005 ± 0.002**

**Table 3:** Results of Automatic Barcode Gap Discovery (ABGD) analysis.

**Substitution model**	**Partition**	**Prior intraspecific divergence (P)**						
		**0.001**	**0.0017**	**0.0028**	**0.0046**	**0.0077**	**0.0129**	**0.0215**
K2P	Initial	5	5	5	5	5	5	4
	Recursive	32						
JC	Initial	6	6	6	6	6	6	4
	Recursive	32						
SD	Initial	6	6	6	6	6	6	3
	Recursive	11	9	9				

**Notes.**

Substitution models: K2P (Kimura 2-parameters), JC (Jukes and Cantor) and SD (simple distance).

### Coalescent-based species delimitation methods

The bPTP and GMYC methods using cyt-*b* matrix (with sequences from this work plus sequences from GenBank) recognized 45 and 69 candidate species, respectively (Fig. 3). bPTP and GMYC recognized *C. goytaca* and *C. vivoi* as valid species. Conversely, both analyses detected more than one species within the remaining clades, suggesting that *C. marinhus*, *C. maracajuensis*, *C. scotti*, *C. akroai*, *C. langguthi*, and *C. subflavus* are polytypic.

The bPTP and GMYC analyses using concatenated mitochondrial matrix cyt-*b* + COI (with sequences produced in this study) showed more conservative results, yielding 25 and 7 candidate species, respectively (Fig. 4). In bPTP, *C. maracajuensis*, *C. marinhus*, *C. goytaca*, and *C. vivoi* were recovered as single species each while the remaining species were considered complexes (Fig. 4). A different scenario was observed in the GMYC analysis in which *C. maracajuensis* and *C. marinhus* were considered single species each, *C. scotti* and *C. akroai* were recovered as the same putative species as well as *C. vivoi, C. subflavus* and *C. goytaca* that were considered one entity. In contrast, *C. langguthi* was considered polytypic (Fig. 4).

Multi-locus species delimitation method BPP recovered eight lineages as putative species, with high posterior probability: *C. maracajuensis*, *C. marinhus*, *C. scotti*, *C. akroai*, *C. langguthi*, *C. vivoi*, *C. goytaca* and *C. subflavus* (Fig. 4). Coalescent-based STEM analysis was concordant with BPP method, except that the highest support scenario recovered nine species being two species within *C. subflavus*, one represented by samples recovered in the subclade v and the other with the remaining samples (Fig. 4).

Except for the single-locus analyses with cyt-*b* matrix that overestimate the number of candidate species, some agreement can be observed between the coalescent-based analyses. Both *C. macarajuensis* and *C. marinhus* were recovered as two different species in the four methods (bPTP mit, GMYC mit, BPP and STEM). *Cerradomys vivoi* and *C. goytaca* were also observed as single valid species in bPTP, BPP and STEM as well as the *C. subflavus* subclade v in bPTP and STEM. Additionally, more than one species was detected within *C. langguthi* in both bPTP and GMYC (Figs. 3 and 4).

The most congruent result among cytogenetic, ABGD, single-, and multi-locus analyses—corroborated with previous morphologic studies—recognized eight lineages as different species (mean C_*tax*_ = 0.72; Table S5). These lineages correspond to *C. maracajuensis*, *C. marinhus*, *C. scotti*, *C. akroai*, *C. langguthi*, *C. vivoi*, *C. goytaca*, and *C. subflavus*. The most incongruent analysis was bPTP using a mitochondrial data set, which detected 24 species (mean C_*tax*_ = 0.28; Table S5).

### Molecular dating

**Figure 5:**
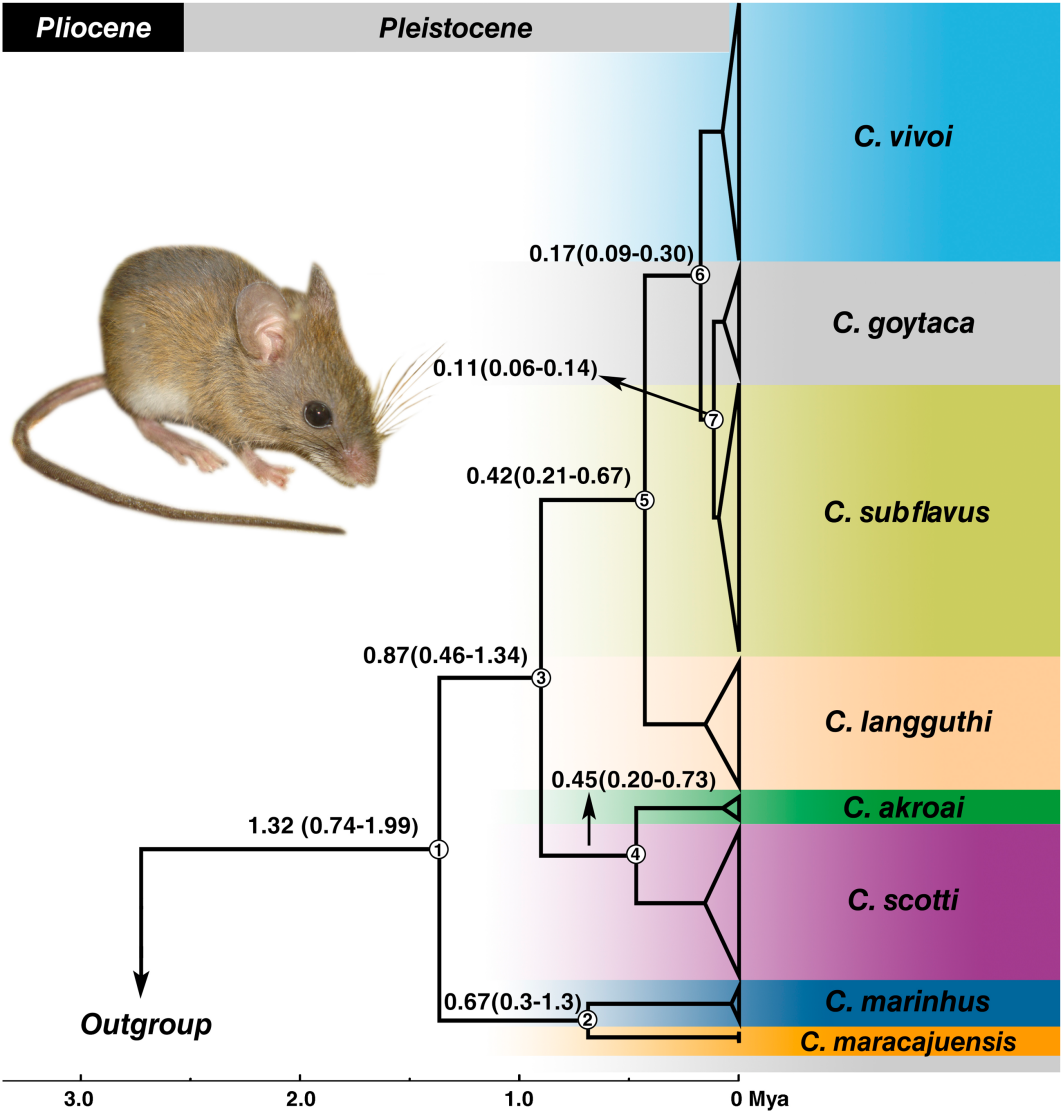
Divergence times estimation of *Cerradomys* obtained from Bayesian analysis of concatenated data set (cyt-*b*, COI, IRBP and i7FBG). Values in the nodes represent the divergence time in millions of years and 95% highest probability density (HPD). Bayesian supports in all clades were 1.0. Node numbers are indicated. Photo: *Cerradomys goytaca* from Parque Nacional Restinga de Jurubatiba, RJ, Brazil.

Estimates of divergence times placed the most recent common ancestor (MRCA) of the genus in the Pleistocene at 1.32 Mya with 95% confidence interval of the highest posterior density (HPD) between 0.74–1.99 Mya (node 1) (Fig. 5) and most of the splits occurred during the medium to late Pleistocene (Fig. 5). *C. marinhus* and *C. maracajuensis* (node 2) split at 0.67 Mya (95% HPD: 0.3–1.3 Mya) and the remaining species (node 3) were estimated at 0.87 Mya (95% HPD: 0.46–1.34 Mya). Node 4 gave rise to the sister species *C. akroai* and *C. scotti* at 0.45 Mya (95% HPD: 0.2–0.73 Mya), while node 5, estimated at 0.42 Mya (95% HPD: 0.21–0.67 Mya), corresponds to MRCA of the species *C. langguthi, C. vivoi, C. goytaca*, and *C. subflavus*. *C. vivoi* diverged from *C. subflavus* and *C. goytaca* at 0.17 Mya (0.09–0.3 Mya) (node 6). Divergence time between *C. subflavus* and *C. goytaca* (node 7) lineages was estimated at 0.11 (0.06–0.14 Mya) (Fig. 5).

## Discussion

### *Cerradomys* species limits

This is the first study that uses chromosomal data together with multiple molecular approaches (multi-locus phylogenetic inference, DNA barcoding, coalescent-based species delimitation, and divergence time estimates) to access species delimitation and patterns and processes of differentiation in the genus *Cerradomys* using a large sample, increasing geographically the coverage of the genus.

Considering the congruence among the distinct methods applied (Carstens et al., 2013), our results support the eight *Cerradomys* species as the most concordant scenario (mean C_*tax*_: 0,74; Table S5). Hence, *C. maracajuensis*, *C. marinhus*, *C. scotti*, *C. akroai*, *C. langguthi*, *C. vivoi*, *C. goytaca,* and *C. subflavus* are valid species although *C. langguthi* and *C. subflavus* may represent more than one taxonomic entity corroborated by at least two approaches.

### Discordance among lines of evidence

Cytogenetic data can distinguish the eight nominal taxa since each one of them has its karyotype (Fig. 2), although Robertsonian rearrangements and pericentric inversion were described for *C. langguthi* and *C. subflavus* (Figs. 2H–2J); (Maia & Hulak, 1981; Almeida & Yonenaga-Yassuda, 1985; Di-Nizo, Ferguson-Smith & Silva, 2020). Even in the species that share the same diploid number, the fundamental number, size and morphology of the chromosomes can accurately discriminate them (*e.g.*: *C. maracajuensis*, *C. marinhus* and *C. subflavus*—2n = 56; *C. vivoi* and *C. langguthi*—2n = 50; *C. subflavus* and *C. goytaca*—2n = 54).

Molecular phylogeny did not support the monophyly of all described species since *C. subflavus* was recovered as paraphyletic to *C. goytaca*. Genetic distance analyses using both cyt-*b* and COI data sets resulted in overlapping of interspecific distance between *C. goytaca* and *C. subflavus* (cyt-*b*: 0.7%; COI: 0.9%), and intraspecific distance of *C. langguthi* (cyt-*b* and COI: 1.2%) (Tables 1 and 2). The high intraspecific distance observed in *C. langguthi* when compared to the intraspecific values of the remaining species could be another indicator that *C. langguthi* may be more than one putative species. Conversely, the low interspecific distance between *C. goytaca* and *C. subflavus* could be related to their recent cladogenesis (0.11 mya) and the mitochondrial genes have not accumulated enough mutations.

All the other analyses (cytogenetics, bPTP, GMYC cyt-*b*, BPP, and STEM) recognized *C. vivoi*, *C. goytaca,* and *C. subflavus* as distinct species and detected additionally cryptic species. Thus, previous hypothesis (based only on cyt-*b*) that suggests *C. goytaca* as junior synonym of *C. subflavus* (Bonvicino, Casado & Weksler, 2014) is incongruent with our results—in addition to the morphometric analyses described by Tavares, Pessôa & Seuánez (2016) and to the cytogenetic data reported by Di-Nizo, Ferguson-Smith & Silva (2020) that suggest that hybrids may not be viable.

Coalescent-based analyses suggested that some species may be species-complexes. bPTP and GMYC using cyt-*b* data set clearly inflated the number of species within the genus (Fig. 3; Table 4), probably because those methods can be misidentifying population structure as species delimitation (Hillis, 2019).

**Table 4:** Lines of evidence supporting *Cerradomys* species.

**Morphology**	**Cytogenetic**	**Monophyly**	**ABGD**	**bPTP cyt-** ***b***	**GMYC cyt-** ***b***	**bPTP mit**	**GMYC mit**	**BPP**	**STEM**
*C. maracajuensis*	*C. maracajuensis*	*C. maracajuensis*	*C. maracajuensis*	*C. maracajuensis* (3)	*C. maracajuensis* (2)	*C. maracajuensis*	*C. maracajuensis*	*C. maracajuensis*	*C. maracajuensis*
*C. marinhus*	*C. marinhus*	*C. marinhus*	*C. marinhus*	*C. marinhus* (3)	*C. marinhus* (6)	*C. marinhus*	*C. marinhus*	*C. marinhus*	*C. marinhus*
*C. scotti*	*C. scotti*	*C. scotti*	*C. scotti*	*C. scotti* (15)	*C. scotti* (26)	*C. scotti* (10)	*C. scotti* *C. akroai*	*C. scotti*	*C. scotti*
*C. akroai*	*C. akroai*	*C. akroai*	*C. akroai*	*C. akroai* (2)	*C. akroai* (3)	*C. akroai* (2)		*C. akroai*	*C. akroai*
C. langguthi^*^	C. langguthi^**^	*C. langguthi*	*C. langguthi*	*C. langguthi* (10)	*C. langguthi* (10)	*C. langguthi* (5)	*C. langguthi* (3)	*C. langguthi*	*C. langguthi*
C. vivoi^*^	*C. vivoi*	*C. vivoi*	*C. vivoi* *C. goytaca* *C. subflavus*	*C. vivoi*	*C. vivoi*	*C. vivoi*	*C. vivoi* *C. goytaca* *C. subflavus*	*C. vivoi*	*C. vivoi*
*C. goytaca*	*C. goytaca*	*C. goytaca*		*C. goytaca*	*C. goytaca*	*C. goytaca*		*C. goytaca*	*C. goytaca*
*C. subflavus*	C. subflavus^**^			*C. subflavus* (10)	*C. subflavus* (20)	*C. subflavus* (4)		*C. subflavus*	*C. subflavus* (2)
**8 CS**	**8 CS**	**7 CS**	**6 CS**	**45 CS**	**69 CS**	**25 CS**	**7 CS**	**8 CS**	**9 CS**

**Notes.**

CS, Candidate species.

* *C. langguthi* and *C. vivoi* may overlap quantitative and qualitative morphological characteristics in populations that occur in the intersection of their distribution.

** *C. langguthi* and *C. subflavus* present chromosomal polymorphisms.

Single-locus bPTP and GMYC (with cyt-*b* and mitochondrial data sets) revealed more than one species within *C. langguthi* and this species, as observed before, presented the highest cyt-*b* and COI K2P intraspecific distances (1.2%). Although chromosome polymorphism was observed in *C. langguthi* (Maia & Hulak, 1981), the only karyotype observed in our sample (2n = 46, FN = 56) was present in all subclades and morphological data is not performed for the entire sample.

Besides, bPTP (cyt-*b* and mitochondrial data sets), GMYC (cyt-*b* data set), and STEM, together with the polytomy recovered in the phylogenetic analyses, suggest that *C. subflavus* may be composed of cryptic species. Some of the candidate species recovered within *C. subflavus* in the coalescent-based analyses coincide with subclades i, iii and v recovered by molecular phylogeny. Although this species presents chromosome polymorphisms, the three karyotypes were found in the same subclade (v).

Thus, the chromosome variation observed in *C. langguthi* and *C. subflavus* is consistent with the hypothesis that they may be incipient species and that what is being called now as polymorphisms, is part of an ongoing process of speciation.

We suggest that a taxonomic revision should be performed in *C. langguthi* and *C. subflavus*, with exhaustive sampling covering its entire distribution, to evaluate if these are cases of species-complexes that underwent chromosome polymorphisms and molecular evolution before being split in different species, since molecular times of divergence showed a recent diversification (<0.17 Mya; Fig. 5).

Discordances among lines of evidence are expected and reflect the disconnection of character evolution due to faster divergence in some characters than in others (Orr & Smith, 1998; Smith et al., 2005; Lougheed et al., 2006). Rapid adaptive radiation can result in morphologically divergent species with low levels of molecular differentiation (Padial et al., 2009), which should be the case of *C. goytaca* and *C. subflavus*. Moreover, these species have allopatric distributions (the former is endemic to the Restinga and *C. subflavus* is distributed in other habitats of Atlantic forest and Cerrado, Fig. 1) and they could be under different selective pressures, which can lead to the accumulation of genetic changes over time. Morphology and chromosomes have already differentiated *C. goytaca* and *C. subflavus*, while haplotypes did not reach monophyletism in the case of samples assigned to *C. subflavus*, generating a mismatch between data, even using different and unlinked loci. These incongruent results would be expected since each character evolves at different rates.

In Addition, it is known that incomplete lineage sorting, selection or distinct mutation rates in specific sequences can lead to incongruences among gene trees and consequently among inferences from other characters (Jennings & Edwards, 2005; Pollard et al., 2006; Syring et al., 2007).

### Phylogenetic relationships and patterns of diversification

The monophyly of the genus was recovered and relationships among species have a robust support, congruent with previous studies (Bonvicino & Moreira, 2001; Percequillo, Weksler & Costa, 2011; Bonvicino, Casado & Weksler, 2014; Tavares, Pessôa & Seuánez, 2016). Divergence time estimates here were similar to the one described by Percequillo et al. (2021) that detected the origin of *Cerradomys* in Pleistocene and more recent than the times estimated by Tavares, Pessôa & Seuánez (2016), in which *Cerradomys* gave rise in Pliocene. This difference could be related to the fossils constraints used, because we used a concatenated data set with four genes, and also included sequences of *C. akroai*, not included in the molecular dating published by Tavares, Pessôa & Seuánez (2016).

Within the tribe Oryzomyini, *Cerradomys* belongs to clade D (Weksler, Percequillo & Voss, 2006), a clade with a complex distribution pattern (Prado & Percequillo, 2013) and which the ancestor probably originated in the east of the Andes (Percequillo et al., 2021). Basal splits within this clade recovered *Drymoreomys* (Percequillo, Weksler & Costa, 2011) (east Andes genus) and *Eremoryzomys* (Weksler, Percequillo & Voss, 2006) (an Andes genus) followed by the remaining genera whose ancestor was probably located on Central South America, from which independent dispersal of extant genera including *Cerradomys* towards west occurred (Percequillo et al., 2021).

The Cerrado domain is the area in which the majority of the *Cerradomys* species inhabit. Divergence time estimates obtained here revealed a recent diversification of *Cerradomys* species during the Pleistocene, with the split of the Cerrado species: *C. maracajuensis* (which ranges to the limit between Cerrado and Amazon) and *C. marinhus* about 0.67 Mya, and the remaining species diverged approximately about 0.87 Mya. The split of *C. akroai* and *C. scotti*, both species that also occupy Cerrado, occurred about 0.45 Mya.

The diversification of the other species was estimated in 0.42 Mya, leading to *C. langguthi* in Cerrado, Caatinga (including Brejos) and Atlantic Forest, in the left bank of São Francisco River, northeast Brazil. Approximately 0.17 Mya, occurred the split of *C. vivoi*, which is distributed in Caatinga, Cerrado and transition areas of Atlantic Forest of Sergipe, Bahia, Minas Gerais and São Paulo (new record herein –see below). Finally, the lineages of *C. subflavus* and *C. goytaca* have recently diverged (about 0.11 Mya), and as mentioned above, *C. subflavus* occurs in Cerrado and Atlantic forest, while *C. goytaca* is limited to Restinga of north Rio de Janeiro and south Espírito Santo States.

Percequillo, Hingst-Zaher & Bonvicino (2008) suggested that the São Francisco River represent a barrier for *Cerradomys* species, as *C. langguthi* is restricted to the left bank of this river while *C. vivoi, C. subflavus* and *C. goytaca* are distributed in the right bank. The cladogenesis of *C. langguthi* occurred in the late Pleistocene (Fig. 5) while the changes in the course of São Francisco River to its current position (reaching the Atlantic Ocean on the east coast of Brazil) was estimated in the middle Pleistocene (Mabesoone, 1994).

Tavares, Pessôa & Seuánez (2016) postulated that geographic limit of *C. vivoi*, *C. subflavus* and *C. goytaca* lies between Jequitinhonha and Doce Rivers. However, in this work, *C. vivoi* was found in the upper São Francisco River (locality 58) as well as in São Paulo state (locality 66). Besides, *C. subflavus* was found on both sides of the Doce River (Fig. 1). Despite Atlantic coastal rivers proved to be a gene flow barrier in many taxa of the Neotropical fauna such as lizards (Pellegrino et al., 2005), birds (Silva, Sousa & Castelletti, 2004; Cabanne, Santos & Miyaki, 2007) and small mammals (Ventura et al., 2012; Fegies et al., 2021) our study suggests that rivers may not have been a primary cause of diversification of *Cerradomys*.

Based on the recent divergence times obtained for *Cerradomys*, it is likely that climatic events of the Pleistocene, yielding contraction and expansion of forests, allowed connections between the Atlantic Forest and the Amazon (Costa, 2003; Batalha-Filho et al., 2013; Ledo & Colli, 2017), forming a barrier and interrupting the gene flow between populations that occupied the central open region. In addition, our results corroborate Tavares, Pessôa & Seuánez (2016) showing that the expansion of the ancestral population that gave rise to *C. subflavus* and *C. goytaca* through the Restinga formation may be facilitated by the Cerrado corridor that possibly connected the north of Rio de Janeiro to central Minas Gerais states (Werneck et al., 2012; Machado et al., 2021). Posteriorly, climatic oscillation favoured the expansion of the Atlantic Forest, creating a barrier between the population from Restinga (*C. goytaca*) and the population from Minas Gerais (*C. subflavus*).

Thus, likewise observed for other taxa also predominantly distributed in Cerrado, such as rodents (Almeida, Bonvicino & Cordeiro-Estrela, 2007), primates (Alfaro et al., 2015) and herpetofauna (Machado, Silva & Silva, 2014; Azevedo et al., 2020), the historical events occurred in the Pleistocene (such as Quaternary climatic oscillations) may have played a major role in the diversification of *Cerradomys* or at least in shaping their current distributions. Added to this, and due to the extraordinary karyotypic variation in the group, chromosomal changes in populations isolated by these historical events may have facilitated speciation when these populations came back into contact during periods of forest retreat.

### New distribution records

The large number of samples obtained in this work enhanced the distributional records of two *Cerradomys* species. This is the first record of *C. langguthi* in Atlantic Forest and Brejos of Alagoas state (ESEC Murici and Mata Grande, localities 11 and 12, respectively) and the Cerrado of Piauí state (locality 10) (Fig. 1). Caccavo & Oliveira (2016) have morphologically analyzed samples of *Cerradomys* from Alagoas, but they were not able to identify these individuals since some morphological attributes of *C. langguthi* and *C. vivoi* from this region overlap.

New localities where *C. vivoi* was recorded in this work raised considerably its distribution to the north of São Paulo state (locality 66) and upper São Francisco River (Ibiraba, Bahia state, locality 58) (Fig. 1). The increase in sample coverage obtained in this study was important not only to understand the boundaries of species but also to provide a new hypothesis of diversification, as it was observed that *C. vivoi* is not restricted to the right bank of São Francisco River, but also occurs in the left bank.

Also, *C. scotti* and *C. maracajuensis* were found simpatrically at the Parque Nacional Emas, Goiás state (locality 16), *C. marinhus* and *C. scotti* in Jaborandi, Bahia state (locality 22), and *C. vivoi* and *C. subflavus* in Juramento, Minas Gerais state (locality 50). We also report the occurrence of three *Cerradomys* species (*C. marinhus*, *C. scotti* and *C. subflavus*) in the same locality (23): Uberlândia, Minas Gerais state (Fig. 1).

## Conclusions

The present study integrates cytogenetic information with different molecular analyses using mitochondrial and nuclear data, corroborating the importance of using different approaches to access species limits because of the heterogeneity nature of the characters. Additionally, few studies have applied multispecies coalescent-based methods for the subfamily Sigmodontinae so far.

Herein, we inferred species limits based on cytogenetics, molecular phylogeny and different coalescent approaches. We also provided phylogenetic relationships among *Cerradomys* species and a temporal estimation for their radiation, showing that the climatic events of the Pleistocene shaped the diversity of the genus.

Our study supports that the eight described *Cerradomys* species are valid and suggests that *C. langguthi* and *C. subflavus* may represent complexes with cryptic species that deserves to be investigated deeply including morphology. Data obtained herein, including new distributional records, reiterates that *Cerradomys* and the Neotropical fauna are still poorly known.

## Supplemental Information

10.7717/peerj.13011/supp-1 Supplemental Information 1 Samples analyzed in this work (in bold) and extracted from GenBank Species, Field or Lab and Museum number (when not available, only the museum acronym is mentioned), diploid (2n) and fundamental numbers (FN) when available, GenBank accession numbersand cytogenetics information, and location of the specimens.
N/A: not available. In bold: present study; ^1^Bonvicino, Casado & Weksler (2014); ^2^Tavares, Pessôa & Seuánez (2016); ^3^Bonvicino & Moreira (2001); ^4^Hanson & Bradley (unpublished); ^5^Weksler (2003); ^6^Di-Nizo, Ferguson-Smith & Silva (2020); ^*a*^Holotype; ^*b*^Type locality; ^*c*^Paratype. Acronyms of Brazilian states: Alagoas (AL), Bahia (BA), Ceará (CE), Espírito Santo (ES), Goiás (GO), Mato Grosso (MT), Mato Grosso do Sul (MS), Minas Gerais (MG), Paraíba (PB), Pernambuco (PE), Piaui (PI), Rio de Janeiro (RJ), São Paulo (SP) and Tocantins (TO). Museum, laboratory, locality and collector acronyms: BIO/CIT (Banco de células do laboratório de Citogenética de Vertebrados, IB/USP), CTA (Coleção de tecidos de animais do departamento de Ciências Biológicas, UFES, Brazil), ESEC (Estação Ecológica), LBCE (Laboratório de Biologia e Parasitologia de Mamíferos Silvestres Reservatórios), MCNM (Museu de Ciências Natutal da PUC Minas Gerais), MN (Museu Nacional, UFRJ, Brazil), MUFAL (Museu de História Natural, UFAL, Brazil), NPM (Coleção Biológica do NUPEM/UFRJ), PARNA (Parque Nacional), PE (Parque Estadual), PMP (Estação de Pesquisa, treinamento e educação ambiental Mata do Paraíso), ROD (Laboratório de Ecologia e Evolução), UFPB (Universidade Federal da Paraiba, Brazil). APC (Ana Paula Carmignotto), CEG (Carlos Eduardo Grelle), CRB (Cibele Rodrigues Bonvicino), DPO (Daniele Pedrosa Oliveira), DQM (Diego Queirolo), HGB (Helena de Godoy Bergallo), JFV (Júlio Fernandes Vilela), LG (Lena Geise), LPC (Leonora Pires Costa), MJJS (Maria José de Jesus Silva), MTR (Miguel Trefaut Rodrigues), PHA (Paulo Henrique Asfora), PRG (BRG: Pablo Rodrigues Gonçalves), SLF (Simone Lóss de Freitas), YL (Yuri Leite).

10.7717/peerj.13011/supp-2 Supplemental Information 2 PCR conditions and primers used to amplify mitochondrial (cytochrome *b* –cyt-*b* and cytochrome *c* oxidase subunit I –COI) and nuclear genes (first exon of interphotoreceptor retinoid-binding protein - IRBP and intron 7 of *β*-fibrinogen –i. References: ^1^ Irwin, Kocher & Wilson (1991); ^2^Smith & Patton (1993); ^3^ Folmer et al. (1994); ^4^ Stanhope et al. (1992); ^5^ Matocq, Shurtliff & Feldman (2007).

10.7717/peerj.13011/supp-3 Supplemental Information 3 Matrices constructed for the molecular analyses: genes, number of base pairs, number of terminal taxa and the analyzes performed for each matrix PI: Phylogenetic Inference (Bayesian inference and Maximum Likelihood); SD (species delimitation); PD: pairwise distance; PG: population genetics; and MD: molecular dating.

10.7717/peerj.13011/supp-4 Supplemental Information 4 Outgroups used for molecular dating ^∗^ and phylogenetic analyses ^∗∗^: Species, mitochondrial genes (cyt-*b* and COI), nuclear genes (IRBP and i7FGB) and references (when available)

10.7717/peerj.13011/supp-5 Supplemental Information 5 Taxonomic index of congruence (Ctax) calculated for each pair of approaches. Mean of all the Ctax values (Mean Ctax) and the number of species supported by each approach are indicated The lines of evidence considered were Morphology, Cytogenetics, Automatic Barcoding Gap Discovery (ABGD), bayesian Poisson Tree Processes (bPTP), General Mixed Yule Coalescent model (GMYC), Bayesian Species Delimitation (BPP), and Tree Estimation using Maximum likelihood, (STEM).

10.7717/peerj.13011/supp-6 Supplemental Information 6 Bayesian Inference (BI) tree based on mitochondrial COI gene. Numbers in the nodes indicate BI posterior probability (above 0.50) and ML bootstrap support (above 50), respectively

10.7717/peerj.13011/supp-7 Supplemental Information 7 Bayesian Inference (BI) tree based on nuclear IRBP gene. Numbers in the nodes indicate BI posterior probability (above 0.50) and ML bootstrap support (above 50), respectively

10.7717/peerj.13011/supp-8 Supplemental Information 8 Bayesian Inference (BI) tree based on nuclear i7FBG gene. Numbers in the nodes indicate BI posterior probability (above 0.50) and ML bootstrap support (above 50), respectively

10.7717/peerj.13011/supp-9 Supplemental Information 9 Histogram of pairwise, ranked pairwise distance and number of groups for initial and recursive partitions per value of prior intraespecific divergence, using (A) Kimura 2-parameter distance, (B) Jukes-Cantor and (C) simple distance for the COI gene

## Additional Information and Declarations

### Field Study Permissions

The following information was supplied relating to field study approvals (i.e., approving body and any reference numbers): Field experiments were approved by the Instituto Chico Mendes de Conservação da Biodiversidade (ICMBio) (ICMBio #11603-1 and #24003-4).

### DNA Deposition

The following information was supplied regarding the deposition of DNA sequences: The generated sequences are available at GenBank: MZ273909–MZ273988; MZ434969– MZ435058; MZ389116–MZ389208; MZ404506–MZ404593; MZ984169–MZ984176 (see Table S1).

### Data Availability

The following information was supplied regarding data availability: The raw data are available in a Supplementary File, with species names, field or vouchers number, sequence and cytogenetics information, and location of the specimens.
