# Supplementary material for: Species limits and recent diversification of Cerradomys (Sigmodontinae: Oryzomyini) during the Pleistocene
Source: PeerJ. 2022 Apr 22;10:e13011. doi: 10.7717/peerj.13011 (PMC9037131; doi:10.7717/peerj.13011)
Supplement: Supplemental Information 1 — Species, Field or Lab and Museum number (when not available, only the museum acronym is mentioned), diploid (2n) and fundamental numbers (FN) when available, GenBank accession numbersand cytogenetics information, and location of the specimens. N/A: not available. In bold: present study; 1Bonvicino, Casado & Weksler (2014); 2Tavares, Pessôa & Seuánez (2016); 3Bonvicino & Moreira (2001); 4Hanson & Bradley (unpublished); 5Weksler (2003); 6Di-Nizo, Ferguson-Smith & Silva (2020); aHolotype; bType locality; cParatype. Acronyms of Brazilian states: Alagoas (AL), Bahia (BA), Ceará (CE), Espírito Santo (ES), Goiás (GO), Mato Grosso (MT), Mato Grosso do Sul (MS), Minas Gerais (MG), Paraíba (PB), Pernambuco (PE), Piaui (PI), Rio de Janeiro (RJ), São Paulo (SP) and Tocantins (TO). Museum, laboratory, locality and collector acronyms: BIO/CIT (Banco de células do laboratório de Citogenética de Vertebrados, IB/USP), CTA (Coleção de tecidos de animais do departamento de Ciências Biológicas, UFES, Brazil), ESEC (Estação Ecológica), LBCE (Laboratório de Biologia e Parasitologia de Mamíferos Silvestres Reservatórios), MCNM (Museu de Ciências Natutal da PUC Minas Gerais), MN (Museu Nacional, UFRJ, Brazil), MUFAL (Museu de História Natural, UFAL, Brazil), NPM (Coleção Biológica do NUPEM/UFRJ), PARNA (Parque Nacional), PE (Parque Estadual), PMP (Estação de Pesquisa, treinamento e educação ambiental Mata do Paraíso), ROD (Laboratório de Ecologia e Evolução), UFPB (Universidade Federal da Paraiba, Brazil). APC (Ana Paula Carmignotto), CEG (Carlos Eduardo Grelle), CRB (Cibele Rodrigues Bonvicino), DPO (Daniele Pedrosa Oliveira), DQM (Diego Queirolo), HGB (Helena de Godoy Bergallo), JFV (Júlio Fernandes Vilela), LG (Lena Geise), LPC (Leonora Pires Costa), MJJS (Maria José de Jesus Silva), MTR (Miguel Trefaut Rodrigues), PHA (Paulo Henrique Asfora), PRG (BRG: Pablo Rodrigues Gonçalves), SLF (Simone Lóss de Freitas), YL (Yuri Leite). [file peerj-10-13011-s001.doc]

**Supplementary Table 1.** Samples analyzed in this work (in bold) and extracted from GenBank: Species, Field or Lab and Museum number (when not available, only the museum acronym is mentioned), diploid (2n) and fundamental numbers (FN) when available, GenBank accession numbers of mitochondrial (cyt-*b* and COI) and nuclear genes (IRBP and i7FBG), locality, coordinates, number of the locality plotted on the Figure 1 and references.

| **Species** | **Field #** | **Museum#** | **2n** | **FN** | **Cyt-*b*** | **COI** | **IRBP** | **i7FBG** | **Locality** | **Coordinates** | **Map** | **Reference** |
| --- | --- | --- | --- | --- | --- | --- | --- | --- | --- | --- | --- | --- |
| *C. akroai* | **UUPI137** | **MZUSP30346** | **N/A** | **N/A** | **MZ273969** | **MZ404576** | **MZ435047** | **MZ389154** | **ESECUruçuí-** | **08°52'S;44°58'W** | **1** | **This study** |
|  | **UUPI161** | **MZUSP30347** | 60 | 76 | MT815865 | **MZ404577** | **MZ435048** | **MZ389149** | **Una, PI** |  |  | **This study;** 6 |
|  | **UUPI344** | **MZUSP30348** | **60** | **76** | **MZ273970** | **MZ404575** | **MZ435049** | **MZ389155** |  |  |  | **This study** |
|  | N/A | LBCE12016 | N/A | N/A | KP122210 | N/A | N/A | N/A | Novo Jardim, TO | 11º49’S;46º38’W | 2 | 1 |
|  | N/A | MN80485 | N/A | N/A | KP122214 | N/A | N/A | N/A | TO |  |  | 1 |
|  | N/A | MN80486 | N/A | N/A | KP122215 | N/A | N/A | N/A |  |  |  | 1 |
|  | N/A | MN80491^a^ | N/A | N/A | KP122219 | N/A | N/A | N/A |  |  |  | 1 |
| *C. goytaca* | **PRG2236** | **NPM933** | **54** | **66** | **MZ273961** | **MZ404538** | **MZ435036** | **MZ389148** | **PARNA** | **22°12’S;41°29′W** | **3** | **This study** |
|  | **PRG2240** | **NPM993** | 54 | 66 | MT815868 | **MZ404532** | **MZ435005** | **MZ389128** | **Restinga de** |  |  | **This study;** 6 |
|  | **PRG1860** | **NPM511** | **54** | **66** | **MZ273960** | **MZ404539** | **MZ435004** | **MZ389145** | **Jurubatiba, RJ** |  |  | **This study** |
|  | **PRG1562** | **NPM160** | **N/A** | **N/A** | **MZ273943** | **MZ404540** | **N/A** | **MZ389197** |  |  |  | **This study** |
|  | **PRG1563** | **NPM170** | **N/A** | **N/A** | **MZ273962** | **MZ404537** | **MZ435007** | **MZ389204** |  |  |  | **This study** |
|  | **PRG1586** | **NPM251** | **N/A** | **N/A** | **MZ273963** | **MZ404536** | **MZ435008** | **MZ389185** |  |  |  | **This study** |
|  | **PRG1589** | **NPM254** | **N/A** | **N/A** | **MZ273941** | **MZ404535** | **MZ435034** | **MZ389191** |  |  |  | **This study** |
|  | **PRG1590** | **NPM255** | **N/A** | **N/A** | **MZ273959** | **MZ404534** | **MZ435009** | **MZ389143** |  |  |  | **This study** |
|  | **PRG1861** | **NPM512** | **N/A** | **N/A** | **MZ273944** | **MZ404533** | **MZ435035** | **MZ389194** |  |  |  | **This study** |
|  | **PRG2239** | **NPM992** | **N/A** | **N/A** | **MZ273942** | **MZ404541** | **MZ435037** | **MZ389193** |  |  |  | **This study** |
|  | LBCE-JCM10 | N/A | N/A | N/A | KP122221 | N/A | N/A | N/A | Quissamã, RJ | 22°04′S;41°24′W | 4 | 1 |
|  | LBCE-JCM11 | N/A | N/A | N/A | KP122222 | N/A | N/A | N/A |  |  |  | 1 |
|  | N/A | MN73208 | N/A | N/A | KR149682 | N/A | N/A | N/A |  |  |  | 2 |
|  | N/A | MN73261 | N/A | N/A | KR149679 | N/A | N/A | N/A | São João da Barra, RJ | 21°44′S;41°02′W | 6 | 2 |
|  | N/A | NPM269 | N/A | N/A | KR149680 | N/A | N/A | N/A | Carapebus, RJ^b^ | 22°15′S;41°39′W | 7 | 2 |
|  | N/A | NPM358 | N/A | N/A | KR149677 | N/A | N/A | N/A | Presidente | 21°15′S;40°58′W | 5 | 2 |
|  | N/A | NPM364 | N/A | N/A | KR149678 | N/A | N/A | N/A | Kennedy, ES |  |  | 2 |
| *C. langguthi* | **MTR005** | **MZUSP** | **46** | **56** | **MZ273910** | **MZ404515** | **MZ435025** | **MZ389121** | **Pacoti, CE** | **04º13'S; 38º55'W** | **8** | **This study** |
|  | **MTR011** | **MZUSP** | **46** | **56** | **MZ273917** | **MZ404520** | **MZ435011** | **MZ389119** |  |  |  | **This study** |
|  | **MTR045** | **MZUSP** | **46** | **56** | **MZ273911** | **MZ404512** | **MZ435012** | **MZ389125** |  |  |  | **This study** |
|  | **MTR077** | **MZUSP** | **46** | **56** | **MZ273912** | **MZ404516** | **MZ435013** | **MZ389127** |  |  |  | **This study** |
|  | **LPC289** | **CTA1247** | **N/A** | **N/A** | **MZ273915** | **MZ404518** | **MZ435015** | **MZ389120** | **Crato, CE** | **07°13'S;39°27'W** | **9** | **This study** |
| **Species** | **Field #** | **Museum#** | **2n** | **FN** | **Cyt-*b*** | **COI** | **IRBP** | **i7FBG** | **Locality** | **Coordinates** | **Map** | **Reference** |
| *C. langguthi* | **LPC264** | **CTA1222** | **N/A** | **N/A** | **MZ273987** | **MZ404519** | **MZ435026** | **MZ389124** | **Crato, CE** | **07°13'S;39°27'W** | **9** | **This study** |
|  | **JFV474** | **MN** | 46 | 56 | MT815867 | **MZ404590** | **MZ435010** | **MZ389176** | **Piracuruca, PI** | **04°06'S;41°42'W** | **10** | **This study;** 6 |
|  | **JFV498** | **MN** | **46** | **56** | **MZ273909** | **MZ404514** | **MZ435017** | **MZ389172** |  |  |  | **This study** |
|  | **N/A** | **MUFAL66** | **N/A** | **N/A** | **MZ273918** | **MZ404513** | **MZ435014** | **MZ389174** | **ESEC Murici, AL** | **09º18'S; 35º56'W** | **11** | **This study** |
|  | **N/A** | **MUFAL117** | **N/A** | **N/A** | **MZ273914** | **N/A** | **MZ434971** | **MZ389182** | **Mata Grande, AL** | **09º07’S;37º43'W** | **12** | **This study** |
|  | **N/A** | **MUFAL133** | **N/A** | **N/A** | **MZ273916** | **MZ404517** | **MZ435018** | **MZ389173** |  |  |  | **This study** |
|  | **N/A** | **MUFAL135** | **N/A** | **N/A** | **MZ273913** | **MZ404521** | **MZ434974** | **MZ389175** |  |  |  | **This study** |
|  | N/A | MN69789^a^ | N/A | N/A | AF181276 | N/A | N/A | N/A | João Pessoa, PB | 07º07’S;34º51’W | 13 | 3 |
|  | N/A | LBCE15905 | N/A | N/A | KP122223 | N/A | N/A | N/A | Sousa, PB | 06º45’S;38º14W | 14 | 1 |
|  | PHA282 | N/A | N/A | N/A | KR149696 | N/A | N/A | N/A | Paudalho, PE | 07°52S ;35°09W | 15 | 2 |
| *C.maracajuensis* | **APC618** | **MN71687** | 56 | 58 | MT815864 | **MZ404506** | **N/A** | **MZ389166** | **PARNA Emas,** | **18°15'S;52°53'W** | **16** | **This study;** 6 |
|  | **APC629** | **MN71689** | **56** | **58** | **MZ273985** | **MZ404507** | **MZ435039** | **MZ389163** | **GO** |  |  | **This study** |
|  | N/A | MN44178^a^ | N/A | N/A | AF181278 | N/A | N/A | N/A | Maracaju, MS | 21°38′S, 55°09′W | 17 | 3 |
|  | CRB2790 | N/A | N/A | N/A | KP122224 | N/A | N/A | N/A | São José Xingu, MT | 10°48’S; 52°45W | 18 | 1 |
|  | SVS883 | N/A | N/A | N/A | KP122226 | N/A | N/A | N/A | Campo Verde, MT | 15°29′S;55°09′W | 19 | 1 |
| *C. marinhus* | **ROD340** | **MZUSP** | **56** | **54** | **MZ273982** | **MZ404509** | **MZ435055** | **MZ389165** | **PARNA Veredas,** | **15º14'S; 45º37'W** | **20** | **This study** |
|  | **ROD362** | **MZUSP** | **56** | **54** | **MZ273983** | **MZ404511** | **MZ435056** | **MZ389168** | **MG** |  |  | **This study** |
|  | **CRB1883** | **N/A** | **56** | **54** | **MZ273984** | **MZ404510** | **MZ435058** | **MZ389164** | **Cocos, BA** | **14º10'S; 44º32'W** | **21** | **This study** |
|  | **CRB1835** | **N/A** | 56 | 54 | MT815863 | **MZ404508** | **MZ435057** | **MZ389167** |  |  |  | **This study;** 6 |
|  | N/A | MN63834^c^ | N/A | N/A | AF181279 | N/A | N/A | N/A | Jaborandi, BA | 13º37’S;44º25’W | 22 | 3 |
|  | N/A | LBCE17294 | N/A | N/A | KP122228 | N/A | N/A | N/A | Uberlândia, MG | 18º55’S;48º17’W | 23 | 1 |
| *C. scotti* | **APC552** | **UNB1915** | **58** | **72** | **MZ273986** | **MZ404584** | **MZ435052** | **MZ389169** | **PARNA Emas,** | **18°04'S;52°55'W** | **16** | **This study** |
|  | **APC567** | **MN71671** | **N/A** | **N/A** | **MZ273980** | **MZ404580** | **MZ435051** | **MZ389170** | **GO** |  |  | **This study** |
|  | **APC572** | **UNB1904** | **58** | **72** | **MZ273976** | **MZ404585** | **MZ434972** | **MZ389150** |  |  |  | **This study** |
|  | **APC615** | **MN71686** | **N/A** | **N/A** | **MZ273979** | **MZ404591** | **MZ434987** | **MZ389152** |  |  |  | **This study** |
|  | **MJJS189** | **MZUSP** | 58 | 72 | MT815866 | **N/A** | **MZ435053** | **MZ389159** | **Serra das Galés,** | **16º59’S;50º37’W** | **24** | **This study;** 6 |
|  | **MJJS204** | **MZUSP** | **58** | **72** | **MZ273977** | **MZ404586** | **MZ435054** | **MZ389158** | **GO** |  |  | **This study** |
|  | **MJJS232** | **MZUSP** | **58** | **N/A** | **MZ273974** | **MZ404582** | **MZ435044** | **MZ389160** |  |  |  | **This study** |
|  | **MJJS233** | **MZUSP** | **58** | **N/A** | **MZ273975** | **MZ404581** | **MZ435045** | **MZ389162** |  |  |  | **This study** |
|  | **MJJS255** | **MZUSP** | **N/A** | **N/A** | **MZ273971** | **MZ404579** | **MZ435043** | **MZ389151** |  |  |  | **This study** |
|  | **MJJS260** | **MZUSP** | **N/A** | **N/A** | **MZ273973** | **MZ404587** | **MZ435050** | **MZ389153** |  |  |  | **This study** |
|  | **BIO474** | **N/A** | **N/A** | **N/A** | **MZ273972** | **MZ404583** | **MZ435046** | **N/A** | **PARNA Brasília,GO** | **15º40’S;47º59’W** | **25** | **This study** |
|  | N/A | MN50379 | N/A | N/A | AF181277 | N/A | N/A | N/A | Cavalcante, GO | 13º47’S;47º27’W | 26 | 3 |
|  | N/A | LBCE9446 | N/A | N/A | KP122243 | N/A | N/A | N/A | Aporé, GO | 18º58’S;51º55’W | 27 | 1 |
|  | N/A | LBCE10907 | N/A | N/A | KP122232 | N/A | N/A | N/A |  |  |  | 1 |
|  | N/A | LBCE5848 | N/A | N/A | KP122240 | N/A | N/A | N/A |  |  |  | 1 |
| **Species** | **Field #** | **Museum#** | **2n** | **FN** | **Cyt-*b*** | **COI** | **IRBP** | **i7FBG** | **Locality** | **Coordinates** | **Map** | **Reference** |
| *C. scotti* | N/A | LBCE8749 | N/A | N/A | KP122242 | N/A | N/A | N/A | Campo Alegre de Goiás, GO | 17º38’S; 47º47’W | 28 | 1 |
|  | N/A | LBCE7546 | N/A | N/A | KP122241 | N/A | N/A | N/A | Luziania, GO | 16º15’S;47º57’W | 29 | 1 |
|  | **LPC688** | **CTA1636** | **N/A** | **N/A** | **MZ273978** | **MZ404578** | **MZ435042** | **MZ389157** | **Uberlândia, MG** | **09°05'S;47°56'W** | **23** | **This study** |
|  | N/A | LBCE17287 | N/A | N/A | KP122234 | N/A | N/A | N/A | Campo Florido, MG | 19º45’S;48º34’W | 30 | 1 |
|  | **N/A** | **LBCE4884** | **N/A** | **N/A** | KP122236 | **MZ404588** | **MZ435040** | **MZ389156** | **Aquidauana, MS** | **20°28'S;55°47'W** | **31** | **This study;** 1 |
|  | **N/A** | **LBCE5713** | **N/A** | **N/A** | KP122237 | **MZ404589** | **MZ435041** | **MZ389161** | **Corumbá, MS** | **19°00'S;57°39'W** | **32** | **This study;** 1 |
|  | N/A | LBCE5724 | N/A | N/A | KP122238 | N/A | N/A | N/A |  |  |  | 1 |
|  | N/A | LBCE5725 | N/A | N/A | KP122239 | N/A | N/A | N/A |  |  |  | 1 |
|  | N/A | LBCE11618 | N/A | N/A | KP122233 | N/A | N/A | N/A | Sítio D’Abadia, MS | 14º48’S;46º15’W | 33 | 1 |
|  | N/A | MN61672 | N/A | N/A | KP122244 | N/A | N/A | N/A | Jaborandi, BA | 13º37’S;44º25’W | 22 | 1 |
|  |  |  |  |  |  |  |  |  |  |  |  |  |
|  | CRB2731 | N/A | N/A | N/A | KP122231 | N/A | N/A | N/A | Correntina, BA | 13º20’S;44º37’W | 34 | 1 |
|  | N/A | TK61881 | N/A | N/A | EU579482 | N/A | N/A | N/A | Canindeyui, Paraguay | 24°08′S;55°22′W | 35 | 4 |
| *C. aff. subflavus* | **ROD86** | **MZUSP** | **54** | **62** | **MZ273954** | **N/A** | **N/A** | **MZ389192** | **Guará, SP** | **20°48'S;47°48'W** | **36** | **This study** |
|  | **PCH1461** | **MZUSP** | **N/A** | **N/A** | **MZ273955** | **MZ404543** | **MZ435006** | **MZ389140** |  |  |  | **This study** |
|  | **CIT2053** | **MZUSP** | 56 | 64 | MT815869 | **MZ404545** | **MZ435028** | **MZ389190** | **Itirapina, SP** | **22º15’S;47º49’W** | **37** | **This study;** 6 |
|  | **CIT1396** | **MZUSP** | 55 | 63 | MT815870 | **MZ404544** | **MZ435027** | **MZ389116** | **Rio Claro, SP** | **22º24'S; 47º34'W** | **38** | **This study;** 6 |
|  | **PCH3619** | **MZUSP** | **54** | **62** | **MZ273953** | **MZ404556** | **MZ435016** | **MZ389203** | **São Joaquim da** | **20°34'S;47°51'W** | **39** | **This study** |
|  | **PCH3995** | **MZUSP** | **54** | **62** | **MZ273951** | **MZ404553** | **MZ435038** | **MZ389131** | **Barra, SP** |  |  | **This study** |
|  | **CIT661** | **MZUSP** | **54** | **62** | **MZ273981** | **MZ404529** | **N/A** | **MZ389118** | **Santa Rita do Passa Quatro, SP** | **21º42'S; 47º28'W** | **40** | **This study** |
|  |  |  |  |  |  |  |  |  |  |  |  |  |
|  | **DPO22** | **CTA1802** | **N/A** | **N/A** | **MZ273967** | **MZ404525** | **MZ435001** | **MZ389132** | **Barão de Cocais, MG** | **19°53'S;43°28'W** | **41** | **This study** |
|  | **LPC337** | **CTA1292** | **N/A** | **N/A** | **MZ273956** | **MZ404554** | **MZ434975** | **MZ389196** | **Nova Ponte, MG** | **19°07'S;47°44'W** | **42** | **This study** |
|  | **LPC338** | **CTA1293** | **N/A** | **N/A** | **MZ273957** | **MZ404552** | **MZ435031** | **MZ389139** |  |  |  | **This study** |
|  | **CEG127** | **N/A** | **N/A** | **N/A** | KR149693 | **MZ404551** | **MZ434997** | **MZ389186** | **Nova Lima, MG** | **19°59'S;43°50'W** | **43** | **This study;** 2 |
|  | **LC91** | **CTA1127** | **N/A** | **N/A** | **MZ273966** | **MZ404530** | **MZ435030** | **MZ389144** | **PE Rio Doce, MG** | **19º42’S;42º38’W** | **44** | **This study** |
|  | CEG42 | N/A | N/A | N/A | AF181274 | N/A | N/A | N/A |  |  |  | 3 |
|  | **DQM059** | **N/A** | 54 | 62 | **MZ273988** | **MZ404550** | **MZ435003** | **MZ389135** | **Serra da** | **20°18'S;46°35'W** | **45** | **This study;** 6 |
|  | **DQM006** | **N/A** | **54** | **62** | **MZ273958** | **MZ404555** | **MZ435002** | **MZ389198** | **Canastra, MG** |  |  | **This study** |
|  | **DQM016** | **N/A** | **54** | **62** | **MZ273952** | **MZ404542** | **MZ435000** | **MZ389205** |  |  |  | **This study** |
|  | **YL68** | **CTA934** | **N/A** | **N/A** | **MZ273950** | **MZ404548** | **MZ434998** | **MZ389147** | **São Gonçalo do** | **18°08'S;43°22'W** | **46** | **This study** |
|  | **LC68** | **CTA1104** | **N/A** | **N/A** | **MZ273947** | **MZ404531** | **MZ434999** | **MZ389133** | **Rio Preto, MG** |  |  | **This study** |
| **Species** | **Field #** | **Museum#** | **2n** | **FN** | **Cyt-*b*** | **COI** | **IRBP** | **i7FBG** | **Locality** | **Coordinates** | **Map** | **Reference** |
| *C. aff. subflavus* | N/A | MCNM1242 | N/A | N/A | KR149690 | N/A | N/A | N/A | Brasilândia de Minas, MG | 16°56′S;45°58′W | 47 | 2 |
|  | BRG160 | N/A | N/A | N/A | KR149691 | N/A | N/A | N/A | Araponga, MG | 20°41′S;42°27′W | 48 | 2 |
|  | N/A | LBCE17255 | N/A | N/A | KP122247 | N/A | N/A | N/A | Uberlandia, MG | 18º55’S;48º17’W | 23 | 1 |
|  | N/A | LBCE17293 | N/A | N/A | KP122248 | N/A | N/A | N/A |  |  |  |  |
|  | **PMP062** | **MZUFV2800** | **N/A** | **N/A** | **MZ273964** | **MZ404527** | **MZ435032** | **MZ389141** | **Viçosa, MG** | **20°45'S;42°52'W** | **49** | **This study** |
|  | **PMP083** | **MZUFV2805** | **N/A** | **N/A** | **MZ273965** | **MZ404528** | **MZ435033** | **MZ389138** |  |  |  | **This study** |
|  | **PMP109** | **MZUFV2911** | **N/A** | **N/A** | **MZ273968** | **MZ404549** | **MZ434996** | **MZ389137** |  |  |  | **This study** |
|  | **SLF102** | **CTA109** | **N/A** | **N/A** | **MZ273945** | **MZ404523** | **MZ434991** | **MZ389142** | **Nova Viçosa, BA** | **17°53'S;39°26'W** | **52** | **This study** |
|  | **SLF104** | **CTA110** | **N/A** | **N/A** | **MZ273946** | **MZ404524** | **MZ434992** | **MZ389195** |  |  |  | **This study** |
|  | **HGB020** | **N/A** | **N/A** | **N/A** | KR149694 | **MZ404526** | **MZ434994** | **MZ389199** |  |  |  | **This study;** 2 |
|  | **YL492** | **CTA369** | **N/A** | **N/A** | **MZ273949** | **MZ404546** | **MZ435029** | **MZ389136** | **Águia Branca, ES** | **18°52'S;40°49’W** | **53** | **This study** |
|  | **SLF191** | **CTA875** | **N/A** | **N/A** | **MZ273948** | **MZ404547** | **MZ434993** | **MZ389134** | **São Mateus, ES** | **18°52'S;39°51’W** | **54** | **This study** |
| *C. vivoi* | **LG0468** | **N/A** | **N/A** | **N/A** | KR149685 | **MZ404565** | **MZ434979** | **MZ389189** | **Joaíma, MG** | **16°39'S;41°03’W** | **55** | **This study;** 2 |
|  | **LG0471** | **N/A** | **N/A** | **N/A** | **MZ273930** | **MZ404573** | **MZ434980** | **MZ389202** |  |  |  | **This study** |
|  | **LG0487** | **N/A** | **N/A** | **N/A** | **MZ273935** | **MZ404593** | **MZ435024** | **MZ389183** |  |  |  | **This study** |
|  | **LG0416** | **N/A** | **N/A** | **N/A** | **MZ273929** | **MZ404572** | **MZ434984** | **MZ389184** | **Itinga, MG** | **16°35'S;41°46’W** | **56** | **This study** |
|  | **LG0420** | **N/A** | **N/A** | **N/A** | **MZ273937** | **MZ404569** | **MZ434985** | **MZ389178** |  |  |  | **This study** |
|  | **LG0421** | **N/A** | **N/A** | **N/A** | **MZ273927** | **N/A** | **MZ435020** | **MZ389179** |  |  |  | **This study** |
|  | N/A | MN61666 | N/A | N/A | KP122249 | N/A | N/A | N/A | Juramento, MG | 16°51′S;43°35′W | 50 | 1 |
|  | **LPC225** | **CTA1185** | **N/A** | **N/A** | **MZ273933** | **MZ404560** | **MZ434973** | **MZ389123** | **Andaraí, BA** | **12°48'S;41°15’W** | **51** | **This study** |
|  | **LPC212** | **CTA1172** | **N/A** | **N/A** | **MZ273920** | **MZ404558** | **MZ434969** | **MZ389130** |  |  |  | **This study** |
|  | **N/A** | **LBCE1501** | **N/A** | **N/A** | **MZ273925** | **MZ404567** | **MZ434976** | **MZ389207** | **Caetité, BA** | **14°04'S;42°28’W** | **57** | **This study** |
|  | **N/A** | **LBCE1504** | **N/A** | **N/A** | **MZ273921** | **MZ404563** | **MZ434983** | **MZ389208** |  |  |  | **This study** |
|  | **CIT357** | **N/A** | **50** | **64** | **MZ273936** | **MZ404559** | **MZ435021** | **MZ389122** | **Ibiraba, BA** | **10º53'S;43º05’W** | **58** | **This study** |
|  | LG211 | N/A | N/A | N/A | KR149688 | N/A | N/A | N/A | Itaetê, BA | 12°58′S;40°57′W | 59 | 2 |
|  | N/A | MN35898^a^ | N/A | N/A | AF181275 | N/A | N/A | N/A | Itabuna, BA | 14º47’S;39º16’W | 60 | 3 |
|  | **CD002** | **N/A** | **N/A** | **N/A** | **MZ273922** | **MZ404592** | **MZ434995** | **MZ389206** | **Lençóis, BA** | **12°33'S;41°23’W** | **61** | **This study** |
|  | **CD003** | **N/A** | **N/A** | **N/A** | **MZ273931** | **MZ404568** | **MZ435022** | **MZ389171** |  |  |  | **This study** |
|  | **CD132** | **N/A** | **N/A** | **N/A** | **MZ273932** | **N/A** | **MZ434981** | **MZ389177** |  |  |  | **This study** |
|  | **CD133** | **N/A** | **N/A** | **N/A** | **MZ273919** | **MZ404562** | **MZ434986** | **MZ389181** |  |  |  | **This study** |
|  | **CIT1264** | **N/A** | **50** | **64** | **MZ273939** | **MZ404574** | **MZ434970** | **MZ389117** | **Morro do** | **11º36'S;41º38’W** | **62** | **This study** |
|  | N/A | MN75905 | N/A | N/A | KR149684 | N/A | N/A | N/A | **Chapéu, BA** |  |  | 2 |
|  | **LG0372** | **N/A** | **N/A** | **N/A** | **MZ273926** | **MZ404564** | **MZ435019** | **MZ389201** | **Mucugê, BA** | **13°00'S;41°22’W** | **63** | **This study** |
|  | **LG0318** | **N/A** | **N/A** | **N/A** | **MZ273934** | **MZ404570** | **MZ434977** | **MZ389188** |  |  |  | **This study** |
|  | **LG0325** | **N/A** | **N/A** | **N/A** | KR149687 | **MZ404571** | **MZ434978** | **MZ389200** |  |  |  | **This study;** 2 |
|  | **YL208** | **CTA994** | **N/A** | **N/A** | **MZ273928** | **MZ404566** | **MZ434990** | **MZ389187** | **Serrinha, BA** | **12°43'S;39°05’W** | **64** | **This study** |
|  | **YL220** | **CTA1006** | **N/A** | **N/A** | **MZ273923** | **MZ404557** | **MZ434989** | **MZ389146** |  |  |  | **This study** |
| **Species** | **Field #** | **Museum#** | **2n** | **FN** | **Cyt-*b*** | **COI** | **IRBP** | **i7FBG** | **Locality** | **Coordinates** | **Map** | **Reference** |
| *C. vivoi* | **LC104** | **CTA1137** | **N/A** | **N/A** | **MZ273938** | **MZ404522** | **MZ435023** | **MZ389129** | **Una, BA** | **15°21'S;39°00’W** | **65** | **This study** |
|  | **LPC114** | **CTA1147** | **N/A** | **N/A** | **MZ273924** | **MZ404561** | **MZ434988** | **MZ389126** |  |  |  | **This study** |
|  | **CIT 1472** | **N/A** | **50** | **64** | **MZ273940** | **N/A** | **MZ434982** | **MZ389180** | **Ribeirão Preto, SP** | **21º08'S;47º49’W** | **66** | **This study** |

N/A: not available. In bold: present study; ^1^Bonvicino, Casado & Weksler (2014); ^2^Tavares, Pessôa & Seuánez (2016); ^3^Bonvicino & Moreira (2001); ^4^Hanson & Bradley (unpublished); ^5^Weksler (2003); ^6^Di-Nizo, Ferguson-Smith & Silva (2020); ^a^Holotype; ^b^Type locality; ^c^Paratype. Acronyms of Brazilian states: Alagoas (AL), Bahia (BA), Ceará (CE), Espírito Santo (ES), Goiás (GO), Mato Grosso (MT), Mato Grosso do Sul (MS), Minas Gerais (MG), Paraíba (PB), Pernambuco (PE), Piaui (PI), Rio de Janeiro (RJ), São Paulo (SP) and Tocantins (TO). Museum, laboratory, locality and collector acronyms: BIO/CIT (Banco de células do laboratório de Citogenética de Vertebrados, IB/USP), CTA (Coleção de tecidos de animais do departamento de Ciências Biológicas, UFES, Brazil), ESEC (Estação Ecológica), LBCE (Laboratório de Biologia e Parasitologia de Mamíferos Silvestres Reservatórios), MCNM (Museu de Ciências Natutal da PUC Minas Gerais), MN (Museu Nacional, UFRJ, Brazil), MUFAL (Museu de História Natural, UFAL, Brazil), NPM (Coleção Biológica do NUPEM/UFRJ), PARNA (Parque Nacional), PE (Parque Estadual), PMP (Estação de Pesquisa, treinamento e educação ambiental Mata do Paraíso), ROD (Laboratório de Ecologia e Evolução), UFPB (Universidade Federal da Paraiba, Brazil). APC (Ana Paula Carmignotto), CEG (Carlos Eduardo Grelle), CRB (Cibele Rodrigues Bonvicino), DPO (Daniele Pedrosa Oliveira), DQM (Diego Queirolo), HGB (Helena de Godoy Bergallo), JFV (Júlio Fernandes Vilela), LG (Lena Geise), LPC (Leonora Pires Costa), MJJS (Maria José de Jesus Silva), MTR (Miguel Trefaut Rodrigues), PHA (Paulo Henrique Asfora), PRG (BRG: Pablo Rodrigues Gonçalves), SLF (Simone Lóss de Freitas), YL (Yuri Leite).
