# Supplementary material for: Species limits and recent diversification of Cerradomys (Sigmodontinae: Oryzomyini) during the Pleistocene
Source: PeerJ. 2022 Apr 22;10:e13011. doi: 10.7717/peerj.13011 (PMC9037131; doi:10.7717/peerj.13011)
Supplement: Supplemental Information 2 — References: 1 Irwin, Kocher & Wilson (1991); 2Smith & Patton (1993); 3 Folmer et al. (1994); 4 Stanhope et al. (1992); 5 Matocq, Shurtliff & Feldman (2007). [file peerj-10-13011-s002.doc]

**Supplementary Table 2.** PCR conditions and primers used to amplify mitochondrial (cytochrome *b* – cyt-*b* and cytochrome *c* oxidase subunit I – COI) and nuclear genes (first exon of interphotoreceptor retinoid-binding protein - IRBP and intron 7 of β-fibrinogen – i7FGB) and best-fit models and partitions obtained for each gene.

| **Gene** | **Primers** | **References** | **Initial Denaturation** | **# Cycles** | **Cycles** | | | **Final Extension** | **Best-fit models** |
| --- | --- | --- | --- | --- | --- | --- | --- | --- | --- |
|  |  |  |  |  | **Denaturation** | **Annealing** | **Extension** |  |  |
| Cyt-*b* | MVZ05  MVZ16 | 1  2 | 94°C/5 min | 39 | 94°C/30sec | 48°C/45sec | 72°C/45sec | 72°C/5min | 1^st^ codon: F81+I |
|  |  |  |  |  |  |  |  |  | 2^nd^ codon: GTR+G |
|  |  |  |  |  |  |  |  |  | 3^rd^ codon: K80+I+G |
| COI | LCO1490  HCO2198 | 3 | 94ºC/5 min | 39 | 94°C/30sec | 44ºC/45sec | 72ºC/45sec | 72ºC/5min | 1^st^ codon: K80+I+G |
|  |  |  |  |  |  |  |  |  | 2^nd^ codon: F81+I+G |
|  |  |  |  |  |  |  |  |  | 3^rd^ codon: GTR+G |
| IRBP | A1  F | 4 | 94ºC/5 min | 39 | 94°C/30sec | 62ºC/45sec | 72ºC/3min | 72ºC/5min | 1^st^ and 2^nd^ codons: K80 |
|  |  |  |  |  |  |  |  |  | 3^rd^ codon: K80+I+G |
| i7FGB | Β17-mammL  Βfib-mammaU | 5 | 94ºC/5 min | 39 | 94°C/30sec | 60ºC/90sec | 72°C/60sec | 72ºC/5min | K80+I+G |

References:^1^ Irwin, Kocher & Wilson (1991); ^2^Smith & Patton (1993); ^3^ Folmer et al. (1994); ^4^ Stanhope et al*.* (1992); ^5^ Matocq, Shurtliff & Feldman (2007).
