# Supplementary material for: Species limits and recent diversification of Cerradomys (Sigmodontinae: Oryzomyini) during the Pleistocene
Source: PeerJ. 2022 Apr 22;10:e13011. doi: 10.7717/peerj.13011 (PMC9037131; doi:10.7717/peerj.13011)
Supplement: Supplemental Information 3 — PI: Phylogenetic Inference (Bayesian inference and Maximum Likelihood); SD (species delimitation); PD: pairwise distance; PG: population genetics; and MD: molecular dating. [file peerj-10-13011-s003.doc]

**Supplementary Table 3**. Matrices constructed for the molecular analyses: genes, number of base pairs, number of terminal taxa (excluding outgroup) and the analyses performed for each matrix.

|  | **Matrix** | **# bp** | **# taxa** | **Analyses** |
| --- | --- | --- | --- | --- |
| **I** | Cyt-*b* | 733 bp | 135 (80 this work + 55 GenBank) | PI, single-locus SD and PD |
| **II** | COI | 616 bp | 90 (this work) | PI, PD and ABGD |
| **III** | IRBP | 790 bp | 90 (this work) | PI and single-locus SD |
| **IV** | i7FBG | 676 bp | 92 (this work) | PI and single-locus SD |
| **V** | mitochondrial (cyt-*b*+COI) | 1349 bp | 93 (this work) | single-locus SD |
| **VI** | multi-locus (cyt-*b*+COI+IRBP+i7FBG) | 2815 bp | 94 (this work) | PI and SD |
| **VII** | multi-locus (cyt-*b*+COI+IRBP+i7FBG) | 2815 bp | 94 (this work) | MD* |

PI: Phylogenetic Inference (Bayesian inference and Maximum Likelihood); SD (species delimitation); PD: pairwise distance; and MD: molecular dating. *For molecular dating, we used the same matrix VI with different outgroups in order to employ fossil calibration.
