# Supplementary material for: Species limits and recent diversification of Cerradomys (Sigmodontinae: Oryzomyini) during the Pleistocene
Source: PeerJ. 2022 Apr 22;10:e13011. doi: 10.7717/peerj.13011 (PMC9037131; doi:10.7717/peerj.13011)
Supplement: Supplemental Information 4 [file peerj-10-13011-s004.doc]

**Supplementary Table 4.** Outgroups used for molecular dating^*^ and phylogenetic analyses^**^: Species, mitochondrial genes (cyt-*b* and COI), nuclear genes (IRBP and i7FGB) and references (when available).

| **Species** | **Cyt-*b*** | **COI** | **IRBP** | **i7FGB** | **Reference** |
| --- | --- | --- | --- | --- | --- |
| *Neotoma mexicana^*^* | MW419114 | HQ980061 | MF097772 | DQ180047 | Hernandez-Canchola, Leon-Paniagua & Esselstyn (2021); Steppan & Schenk (2017); Matocq, Shurtliff & Feldman (2007) |
| *Neotoma bryanti** | DQ781157 | HQ980068 | KC953408 | N/A | Schenk, Rowe & Steppan (2013) |
| *Sigmodon hispidus** | EU073177 | JQ601045 | AY277479 | EU652896 | D’Elía (2003) |
| *Sigmodon alstoni** | EU652907 | HQ919654 | KC953455 | EU652889 | Schenk, Rowe & Steppan (2013) |
| *Reithrodon auritus** | KY754133 | N/A | AY277473 | N/A | Steppan & Schenk (2017); D’Elía (2003) |
| *Akodon mystax** | EF101875 | N/A | MG687422 | MH057707 | Gonçalves et al. (2007; 2020) |
| *Oxymycterus nasutus** | MG687418 | N/A | MG687427 | MH057713 | Gonçalves et al. (2020) |
| *Calomys callosus** | AY033187 | MW009898 | AY277440 | KT361514 | Salazar-Bravo et al. (2002); D’Elía (2003) |
| *Phyllotis darwini** | AY956729 | N/A | N/A | KT383391 | Steppan et al. (2007) |
| *Graomys domorum** | AF159291 | N/A | JQ434411 | N/A | Anderson & Yates (2000); Salazar-Bravo, Pardiñas & D'Elia (2013) |
| *Graomys griseoflavus** | KY753993 | N/A | AY277449 | N/A | Steppan & Schenk (2017); D’Elía (2003) |
| *Oligoryzomys nigripes** | GU126530 | KF815406 | AY163612 | JQ282856 | Percequillo,Weksler & Costa (2011); Vilela et al*.* (2014); Weksler (2003); Agrellos et al. (2012) |
|  |  |  |  |  |  |
| *Calomys tener*** | DQ447300 | GU938935 | JQ434407 | N/A | Almeida, Bonvicino & Cordeiro-Estrela (2007); Muller et al. (2013); Salazar-Bravo, Pardinas & D’Elía (2013) |
| *Holochilus sciureus*** | MZ984169 | N/A | MZ984174 | MZ984175 | CTA2049 - *Present study* |
| *Oligoryzomys flavescens*** | MZ984170 | N/A | MZ984172 | N/A | ROD123 - *Present study* |
