# Supplementary material for: Species limits and recent diversification of Cerradomys (Sigmodontinae: Oryzomyini) during the Pleistocene
Source: PeerJ. 2022 Apr 22;10:e13011. doi: 10.7717/peerj.13011 (PMC9037131; doi:10.7717/peerj.13011)
Supplement: Supplemental Information 5 — The lines of evidence considered were Morphology, Cytogenetics, Automatic Barcoding Gap Discovery (ABGD), bayesian Poisson Tree Processes (bPTP), General Mixed Yule Coalescent model (GMYC), Bayesian Species Delimitation (BPP), and Tree Estimation using Maximum likelihood, (STEM). [file peerj-10-13011-s005.doc]

**Supplementary Table 5.** Taxonomic index of congruence (*Ctax*) calculated for each pair of approaches. Mean of all the *Ctax* values (Mean C*tax*) and the number of species supported by each approach is indicated. The lines of evidence considered were: Morphology, Cytogenetics, Automatic Barcoding Gap Discovery (ABGD), bayesian Poisson Tree Processes (bPTP), General Mixed Yule Coalescent model (GMYC), Bayesian Species Delimitation (BPP), and Tree Estimation using Maximum likelihood, (STEM).

|  | **Paired Ctax** | | | | | | | **Mean Ctax** | **Species number** |
| --- | --- | --- | --- | --- | --- | --- | --- | --- | --- |
|  | **Morphology** | **Cytogenetic** | **ABGD** | **bPTP mit** | **GMYC mit** | **BPP** | **STEM** |
| **Morphology** | - |  |  |  |  |  |  | 0.72 | 8 |
| **Cytogenetic** | 1.00 | - |  |  |  |  |  | 0.72 | 8 |
| **ABGD** | 0.71 | 0.71 | - |  |  |  |  | 0.58 | 6 |
| **bPTP mit** | 0.29 | 0.29 | 0.21 | - |  |  |  | 0.28 | 25 |
| **GMYC mit** | 0.44 | 0.44 | 0.50 | 0.25 | - |  |  | 0.41 | 7 |
| **BPP** | 1.00 | 1.00 | 0.71 | 0.29 | 0.44 | - |  | 0.72 | 8 |
| **STEM** | 0.88 | 0.88 | 0.63 | 0.33 | 0.40 | 0.88 | - | 0.66 | 9 |
