# Supplementary figures and images for: Species limits and recent diversification of Cerradomys (Sigmodontinae: Oryzomyini) during the Pleistocene
Source: PeerJ. 2022 Apr 22;10:e13011. doi: 10.7717/peerj.13011 (PMC9037131; doi:10.7717/peerj.13011)

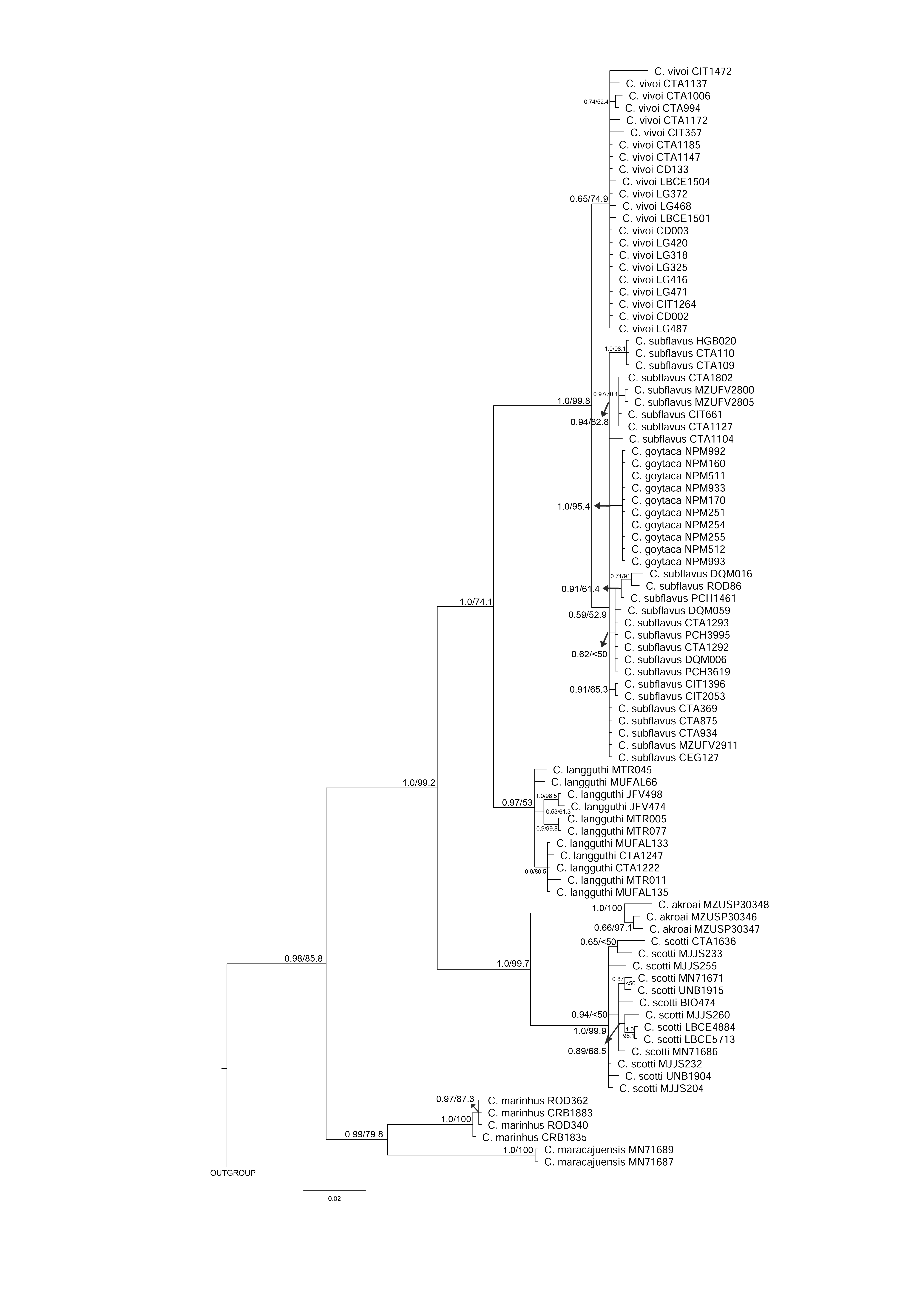

Supplement: Supplemental Information 6 [file peerj-10-13011-s006.jpg]

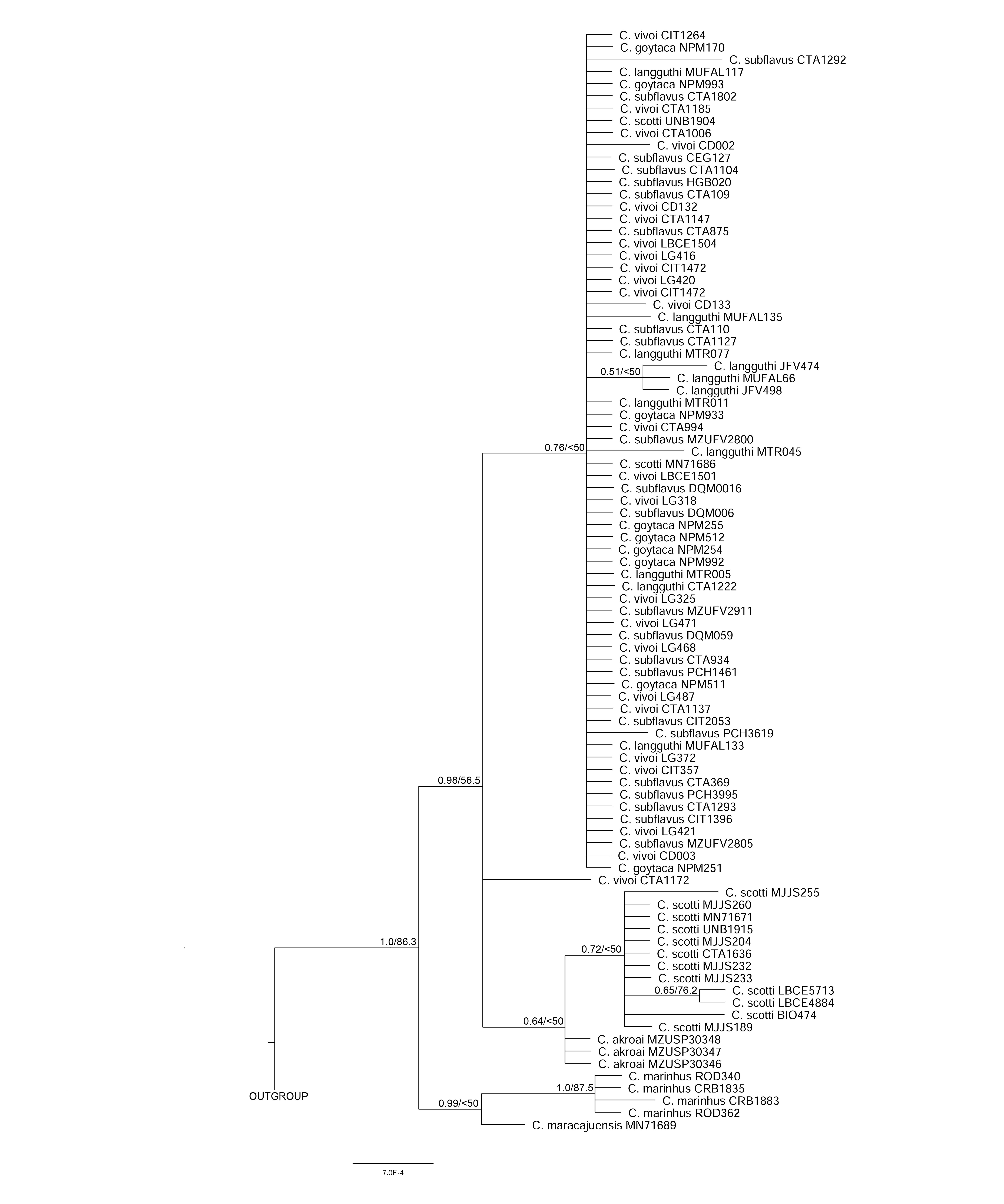

Supplement: Supplemental Information 7 [file peerj-10-13011-s007.jpg]

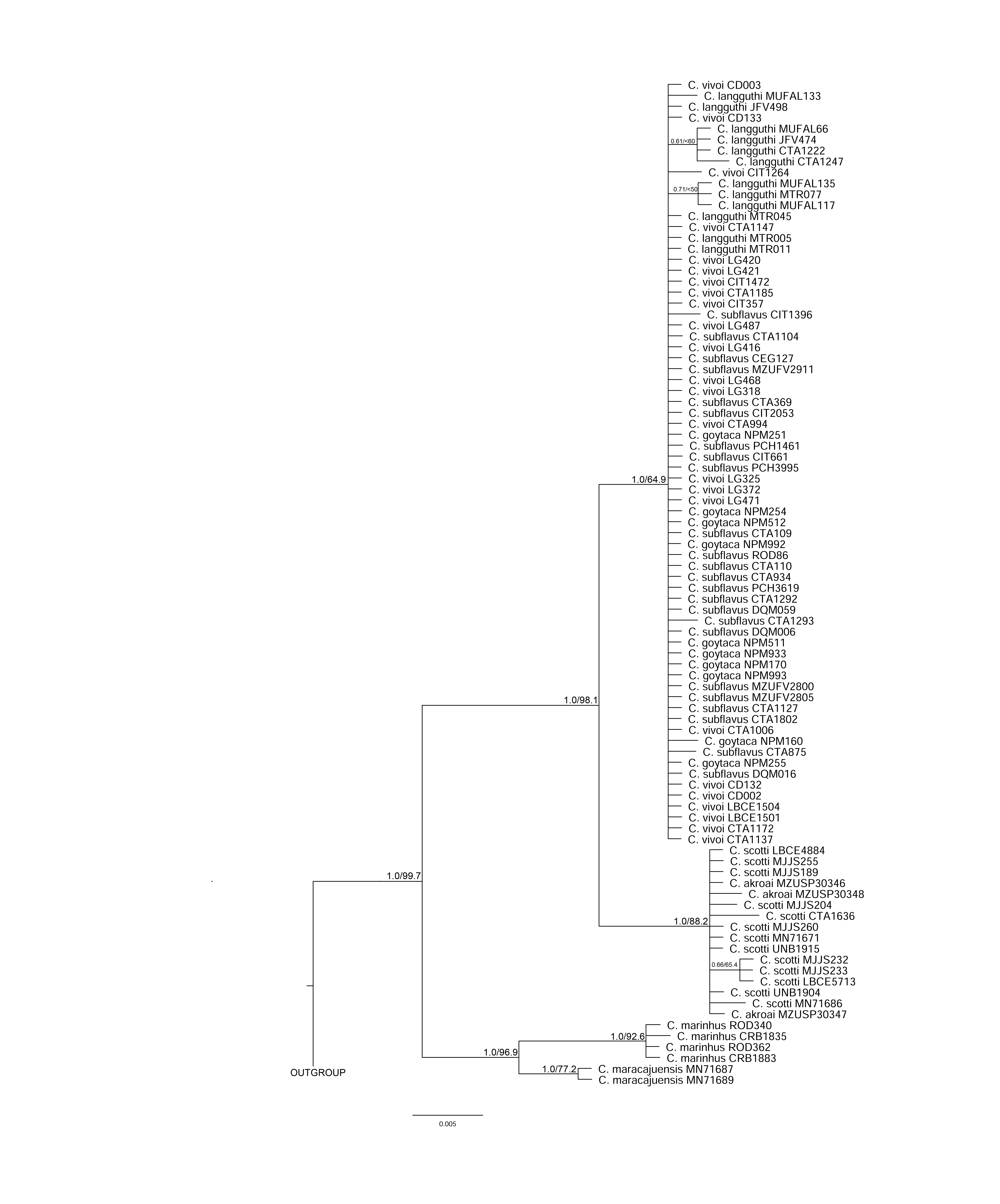

Supplement: Supplemental Information 8 [file peerj-10-13011-s008.jpg]

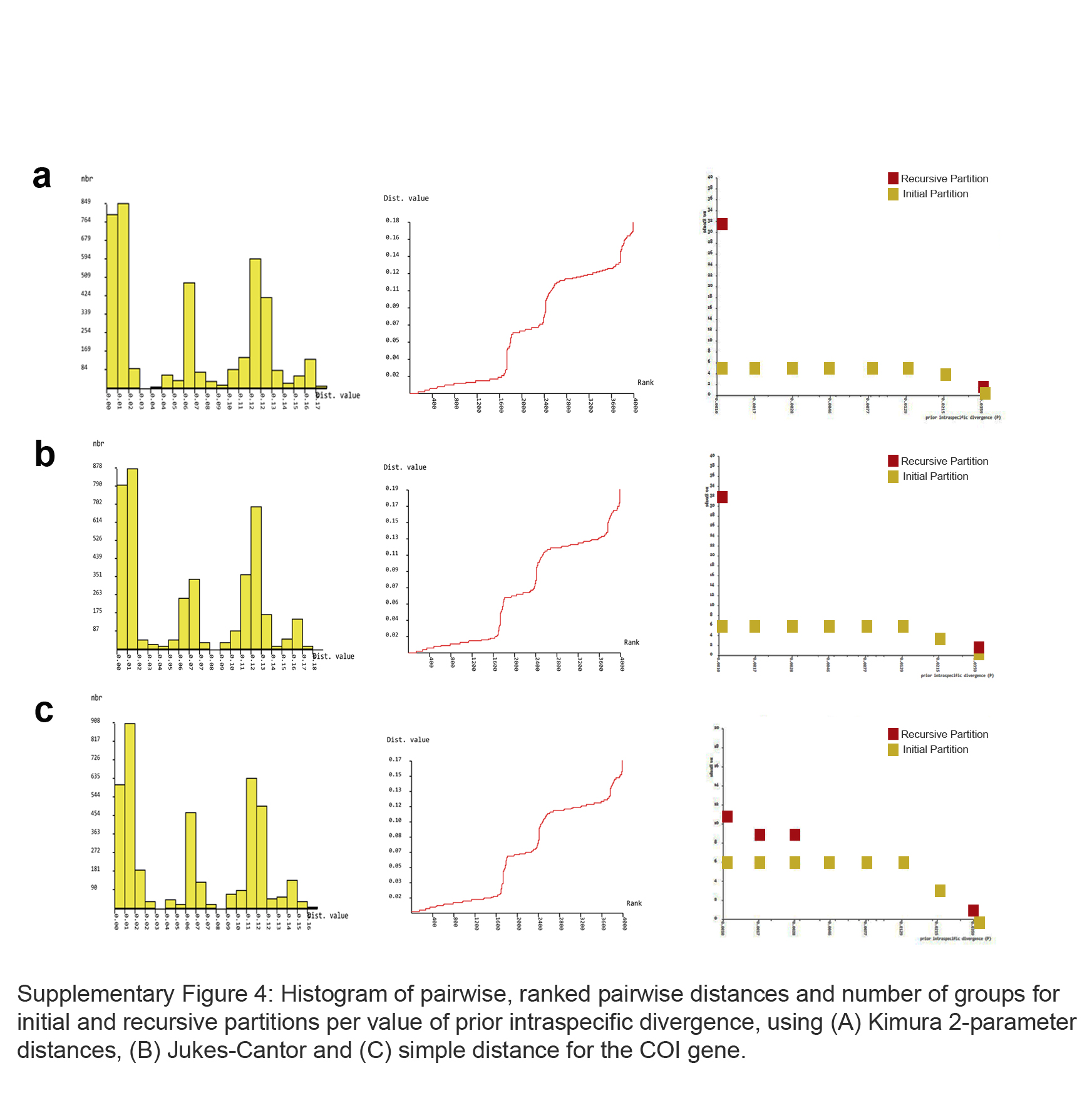

Supplement: Supplemental Information 9 [file peerj-10-13011-s009.jpg]
